# The first step for understanding the molecular mechanism of the antifibrotic effect of inhaling 25(OH)-vitamin D3 and 1,25(OH)_2_-vitamin D3 in the murine model of hypersensitivity pneumonitis

**DOI:** 10.3389/fphar.2025.1610165

**Published:** 2025-08-08

**Authors:** Marta Kinga Lemieszek, Michał Chojnacki, Iwona Paśnik, Alicja Wilczyńska, Wiktoria Gawryś, Jakub Anisiewicz, Ilona Leśniowska, Michał Kiełbus

**Affiliations:** ^1^ Department of Medical Biology, Institute of Rural Health, Lublin, Poland; ^2^ Department of Biochemistry and Molecular Biology, Medical University of Lublin, Lublin, Poland

**Keywords:** pulmonary fibrosis, hypersensitivity pneumonitis, epithelial–mesenchymal transition, calcidiol, calcitriol, animal models

## Abstract

**Introduction:**

Pulmonary fibrosis occurs in several respiratory diseases, among which hypersensitivity pneumonitis (HP) is often ignored in designing therapeutic strategies. We strive to fill these knowledge gaps. Our earlier studies revealed antifibrotic potential of the inhalation of 1,25(OH)2-VD3 and 25(OH)-VD3 based on modulation of the immune response and deposition of extracellular matrix components, both of which are important components of epithelial–mesenchymal transition (EMT), which we focused on in this research. The study aimed to describe the direct impact of VD3-metabolites on EMT in the course of pulmonary fibrosis in HP to understand their therapeutic effect.

**Methods:**

The research was performed in the HP model, wherein pulmonary fibrosis is induced in mice by chronic exposure to antigens of *Pantoea agglomerans* (SE-PA). The study was conducted in VD3-deficient mice, while VD3-sufficient mice were used as the main control. VD3-deficient mice inhaled 100 pg/g of 25(OH)-VD3 or 5 pg/g of 1,25,25(OH)2-VD3 used separately or with SE-PA for 14 days and 28 days. The range of pulmonary fibrosis was determined after Masson trichrome staining. Expression of EMT molecules was examined using real-time PCR and Western blot.

**Results and discussion:**

The studies revealed that VD3-deficiency triggers EMT, the signs of which were increased expression of EMT transcription factors (Snail1, Snail2, Zeb1, and Zeb2), inhibited expression of epithelial cell markers (E-cadherin and occludin), and altered expression of mesenchymal cell markers, including upregulated N-cadherin and vimentin. Pathological changes caused by VD3-deficiencies accelerated in response to SE-PA, the signs of which were: 1) upregulated expression of Snail1, Snail2, Zeb1, Zeb2, Acta2, Cdh2, Fn1, and Vim; 2) downregulated expression of Cdh1 and Ocln; 3) increased level of α-SMA, fibronectin, vimentin, and occludin; 4) decreased amount of N-cadherin; 5) increased deposition of fibers in lung tissue. All negative changes recorded on the transcriptome level in VD3-deficient mice with HP were effectively reduced by inhalations of 25(OH)-VD3 and 1,25,25(OH)2-VD3, suggesting that their antifibrotic effects are associated with EMT inhibition. Nevertheless, the beneficial impact of VD3-metabolites on the proteome level was associated with restoration of the balance in the expression of EMT molecules disturbed by cholecalciferol deficiency and SE-PA exposure; metabolites lowered the overexpressed amounts of fibronectin, vimentin, and occludin and simultaneously increased the expression of vitamin D3 metabolites downregulated N-cadherin and enhanced of vitamin D3 metabolite expression of E-cadherin.

## 1 Introduction

Fibrotic diseases account for one of the most frequent pathologies in aged individuals, involving more than 800 million individuals worldwide (Noble et al., 2012; Pottier et al., 2014). Based on the 2019 Global Burden of Disease (GBD) study, fibrotic disorders caused up to 35% of global deaths in 2019 (GBD, 2019 Diseases and Injuries Collaborators, 2020; Mutsaers et al., 2023). Fibrosis is an excess of fibrous connective tissue production in the area of another type of tissue. Under physiologic conditions (tissue repair and regeneration), deposition of fibrous connective tissue enhances the structure and helps maintain the boundaries of organ compartments. However, an uncontrolled fibrotic process may lead to the loss of the organ’s function. In the lungs, fibrosis eliminates pathologically changed areas of lung tissue from the gas exchange, causing hypoxia and, in advanced cases, death (Ziesche et al., 2013). Thus, pulmonary fibrosis is a progressive and irreversible lung disorder with a strong disadvantageous influence on patients’ quality of life (cough, fatigue, and dyspnea) and short mean life expectancy (3–5 years) (Noble et al., 2012; Ziesche et al., 2013; Ryerson et al., 2014). This situation is detrimental because of the lack of pharmacological therapy—medical interventions targeting lung function improvement and survival enhancement remain disappointing. In contrast, lung transplantation as the only therapeutic option underlines the importance of the problem and its consequences for public health (Lee et al., 2014; Pottier et al., 2014).

Pulmonary fibrosis occurs in several respiratory diseases, including asthma, chronic obstructive pulmonary disease (COPD), idiopathic pulmonary fibrosis (IPF), cystic fibrosis, and hypersensitivity pneumonitis (Ziesche et al., 2013; Spagnolo et al., 2015; Meyer, 2017; Mei et al., 2022; Guo et al., 2022; Ong and Ramsey, 2023; Savin et al., 2023). Despite the different etiology of indicated disorders, a systematically growing number of scientific reports indicate the epithelial-mesenchymal transition (EMT) as a driving force of pulmonary fibrosis (Kim et al., 2006; Ijaz et al., 2014; Hou et al., 2019; Li et al., 2019; Salton et al., 2019; Lemieszek et al., 2020; Mottais et al., 2023).

EMT is a closely coordinated process by which epithelial cells transform into mesenchymal cells. EMT plays an important role during embryogenesis, organ development, and tissue repair. However, EMT also underlies pathological processes, such as cancer genesis and fibrosis. Three types of EMT can be distinguished, among which type 2 is responsible for tissue repair and remodeling (Moustakas and Heldin, 2007; Kalluri and Weinberg, 2009; Wynn and Ramalingam, 2012; Marconi et al., 2021). It must be emphasized that EMT type 2 begins as a part of a regular repair-associated event that generates fibroblasts, myofibroblasts, and other related cells to reconstruct tissues following injury. EMT type 2 is strictly associated with inflammation, which initiates and supervises the healing processes. In the case of physiological repair, EMT ceases once inflammation is attenuated, and it is the signal that the wound is closed. In the case of pulmonary fibrosis, the EMT and inflammation are an ongoing vicious circle, until the fibrotic process reaches the point where it cannot be attenuated by silencing inflammation (Kalluri and Weinberg, 2009; Wynn and Ramalingam, 2012; Ziesche et al., 2013).

The EMT process is triggered by several cellular signaling mechanisms, among which the most important involve transforming growth factor β (TGFβ), a well-known profibrotic factor (Thiery and Sleeman, 2006; Kalluri and Weinberg, 2009; Wynn and Ramalingam, 2012; Marconi et al., 2021). The culmination of signal transduction associated with EMT is the induction of the expression of genes for EMT transcription factors, with the pivotal role of ZEB and Snail families (Kalluri and Weinberg, 2009; Wynn and Ramalingam, 2012; Marconi et al., 2021). Indicated factors modulate the expression of several genes, whose protein products finally disturb epithelial cell polarity, proliferation, and differentiation and release cells from contact inhibition, increasing their motility. The resulting mesenchymal cells are characterized by a higher proliferation rate and invasive capacity. These cells produce a wide range of cytokines, chemokines, and growth factors. They are also responsible for the overproduction and deposition of extracellular matrix components, as well as their further rearrangement (Guarino et al., 2009; Wynn and Ramalingam, 2012; Marconi et al., 2021). Indicated changes are associated with alterations in the expression of several tight junction and adherent junction molecules (*inter alia* E-cadherin, occludin, and claudin are replaced by N-cadherin and fibronectin) as well as cytoskeleton rearrangement (*inter alia* cytokeratin filaments are replaced by vimentin) (Kalluri and Weinberg, 2009; Wynn and Ramalingam, 2012; Marconi et al., 2021).

Because the EMT phenomenon underlies pulmonary fibrosis, the search for effective and safe therapy for this pathology should be based on factors capable of inhibiting this phenomenon. As revealed by several scientific reports, vitamin D3 has great potential in this area because its beneficial impact on EMT is associated with the regulation of the expression of proteins responsible for lung epithelium transformation into mesenchymal cells. Vitamin D3 maintains the proper expression or increases lowered levels of E-cadherin—a key protein for maintaining intercellular connections between epithelial cells (Upadhyay et al., 2013; Fischer and Agrawal, 2014; Jiang et al., 2017; Sari et al., 2020; Xiong et al., 2020; Zheng et al., 2020). To maintain the adhesion of epithelial cells, E-cadherin cooperates with β-catenin. Vitamin D3 inhibits the translocation of β-catenin into the cell nucleus, preventing induction of the expression of genes associated with EMT (Upadhyay et al., 2013; Fischer and Agrawal, 2014; Jiang et al., 2017; Huang et al., 2019; Sari et al., 2020; Xiong et al., 2020; Zheng et al., 2020). In contrast to epithelial markers, vitamin D3 inhibits the expression of mesenchymal markers, such as N-cadherin, responsible for cell migratory capacity, and actin involved in cytoskeleton remodeling (Fischer and Agrawal, 2014; Jiang et al., 2017; Zheng et al., 2020). At the same time, vitamin D3 limits the pathological deposition of extracellular matrix (ECM) components associated with EMT, such as collagen types I, III, and IV (Ramirez et al., 2010; Upadhyay et al., 2013; Fischer and Agrawal, 2014; Jiang et al., 2017; Huang et al., 2019; Tzilas et al., 2019; Zheng et al., 2020; Chang et al., 2021; Zhu et al., 2021), α-SMA (Ramirez et al., 2010; Tan et al., 2016; Huang et al., 2019; Sari et al., 2020; Xiong et al., 2020; Zheng et al., 2020; Chang et al., 2021; Zhu et al., 2021), vimentin (Upadhyay et al., 2013; Fischer and Agrawal, 2014; Jiang et al., 2017; Zheng et al., 2020), and fibronectin (Tzilas et al., 2019; Xiong et al., 2020; Chang et al., 2021). Moreover, vitamin D3 silences the expression of transcription factors Snail1, Snail2, and ZEB1 and thus impacts the expression of EMT target genes (Upadhyay et al., 2013; Fischer and Agrawal, 2014; Jiang et al., 2017; Xiong et al., 2020; Zheng et al., 2020).

As indicated, scientific reports presenting the antifibrotic effect of vitamin D3 based on modulation of EMT originate mainly from cell cultures and animal models predominantly dedicated to IPF. There is a lack of research investigating the impact of vitamin D3 metabolites on the EMT process in hypersensitivity pneumonitis, which is also a common reason for pulmonary fibrosis worldwide (Hyldgaard et al., 2014; Spagnolo et al., 2015; Nogueira et al., 2019). The presented study is the first directly dedicated to this issue. The first suggestion about the ability of vitamin D3 to modulate the EMT phenomenon emerged from the latest publication of our team performed on the murine model of HP (Lemieszek et al., 2024). The mentioned studies revealed that VD3 deficiency significantly increases the pulmonary level of some proteins associated with EMT, for example, fibronectin, collagen type I, FGF2 (fibroblast growth factor 2), and above all, TGFβ1. Moreover, the concentration of indicated proteins additionally elevated in the course of HP (induced by an antigen of *Pantoea agglomerans*) suggested their importance for the development of pulmonary fibrosis observed in this model. Alterations in the expression of proteins associated with EMT noted in VD3-deficient mice with HP were effectively reduced by the animals’ short- and long-term exposure to 25(OH)-VD3 or 1,25,25(OH)_2_-VD3, but a beneficial impact on collagen deposition was noted only after 14 days of VD3 nebulization. The advantageous influence of inhalations of metabolites of vitamin D3 correlated with restoration of a physiological level of calcitriol in both animal serum and lung tissue (Lemieszek et al., 2024). These results were the inspiration for the current study, which is focused on understanding the influence of both 25(OH)-VD3 and 1,25(OH)_2_-VD3 on EMT in the development of pulmonary fibrosis in the murine model of HP.

## 2 Materials and methods

### 2.1 Reagents

Unless otherwise indicated, the chemicals used in the study were purchased from Sigma-Aldrich Co. LLC. The preparation of *Pantoea agglomerans* antigen has been presented previously (Lemieszek et al., 2013; Lemieszek et al., 2021).

### 2.2 Design of study

**FIGURE 1:**
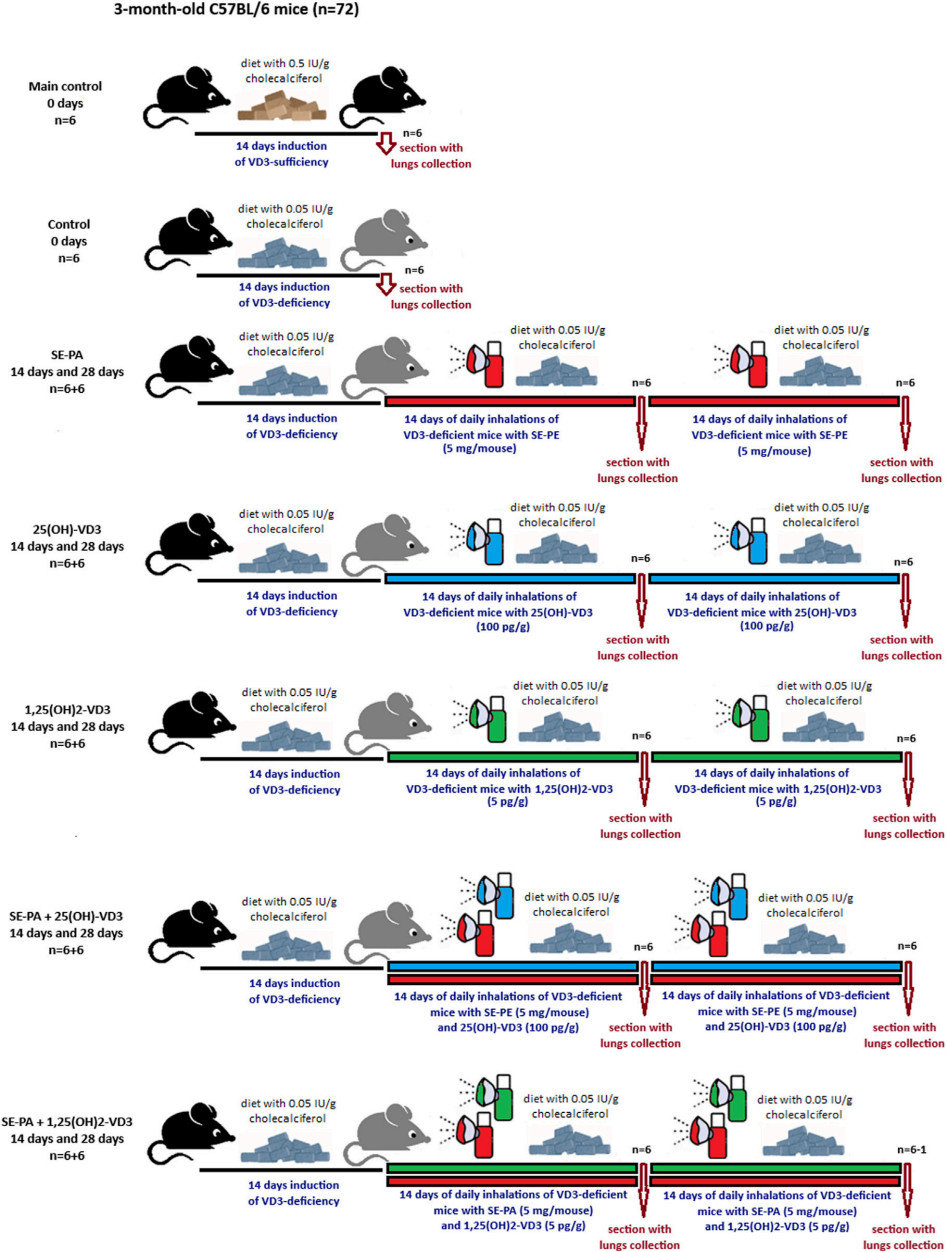
Design of animal study. The research group description is presented in Supplementary Table S1

Three-month-old male C57BL/6 mice were obtained from Mossakowski Medical Research Centre of the Polish Academy of Sciences in Warsaw, Poland (72 animals; mean weight 25.2 g). After arrival at the animal facility, the mice were randomly assigned to research groups. Each group consisted of six mice, which were kept in separate polycarbonate cages under standardized housing conditions (22°C–24°C, natural light–dark cycle) with unlimited access to food and water. The animals, with the exception of those in the main control, were fed a diet with a reduced cholecalciferol level (0.05 IU/g, VD3-deficient diet), while the main control group received feed with a 10-times higher amount of cholecalciferol (standard diet). The indicated rodent feed was obtained from Altromin (Altromin Spezialfutter GmbH & Co. KG, Lage, Germany). The animals received fresh water and feed every day. Experiments were preceded by 1 week of acclimatization, followed by an additional week’s period during which animals were tamed to inhalation chambers and accessories (neck restrainers and nose–mouth silicon masks) by gradually extending the time spent in chambers with dedicated equipment. After acclimatization and adaptation procedures, the actual experiment began. VD3-deficiency was confirmed by lowered calcitriol concentration in both serum and lung tissue compared to animals on a VD3-sufficient diet (Chojnacki et al., 2023; Lemieszek et al., 2024). The mice were exposed to a saline extract of *Pantoea agglomerans* at doses of 5 mg/mouse (SE-PA) or a 25(OH)-VD3 at a concentration of 100 pg/g or 1,25(OH)_2_-VD3 at a concentration of 5 pg/g. Doses of VD3-metabolites were selected based on the results of our earlier studies (Chojnacki et al., 2023; Lemieszek et al., 2024), which revealed that the restoration of the physiological level of calcitriol in the lungs of mice on VD3-defficient diet (0.05 IU/g of cholecalciferol), with or without HP, requires daily exposure to 100 pg/g of 25(OH)-VD3 or 5 pg/g of 1,25 (OH)_2_-VD3 for at least 7 days. The 25(OH)-VD3 and 1,25(OH)2-VD3 were dissolved in 99.8% ethanol to concentrations of 100 ng/mL and 5 ng/mL, respectively. The obtained stock solutions were divided into smaller portions and stored at −20°C. After determining the mass of animals on a given day, the amount of VD3-metabolites necessary to prepare working solutions was calculated. Working solutions directly used for inhalations were obtained by appropriate dilution of the stock solutions in 10 mL of PBS. The final concentration of ethanol in the working solutions was approximately 1.8%. Working solutions were prepared immediately before inhalation. The freeze-dried antigen of *P. agglomerans* was also stored at −20°C. Immediately before inhalation, 30 mg of antigen was dissolved in 10 mL of PBS and directly used for inhalations. The investigated agents were administered to the mice respiratory tracts via nebulization, using the Buxco Inhalation Tower (Data Sciences International, St. Paul, United States) under the following conditions: airflow 2.5 L/min; pressure −0.5 cm H_2_O; room temperature; average nebulization rate 353 μL/min. Inhalations were conducted for half an hour daily for 14 days or 28 days. In the case of research groups exposed simultaneously to both antigen and vitamin D3 metabolite, mice first inhaled SE-PA for 30 min and then inhaled the investigated metabolite for another 30 min. At the end of the experiment (before inhalations or after 14 days or 28 days of inhalations), the mice were sacrificed, followed by lung sample collection for further studies. The graphical design of the study is presented in Figure 1, while the research group description is presented in Supplementary Table S1. The research protocols were approved by the Local Ethics Committee for Animal Experimentation in Lublin, Poland (Resolution Nos. 28/2021, 25/2022, and 73/2022).

### 2.3 Examination of the pulmonary levels of calcitriol and TGFβ1

A detailed description of the preparation of lung tissue homogenates has been presented previously (Lemieszek et al., 2024). The concentration of TGFβ1 in the obtained homogenates was measured using Mouse Transforming Growth Factor Beta 1 (Cloud-Clone Corp., Katy, TX, United States), according to the manufacturer’s instructions. Examination of calcitriol concentration in lung tissue homogenates was preceded by its extraction using the Extraction Kit for 1,25(OH)2 vitamin D ELISA, after which proper investigation using 1,25(OH)2 vitamin D Total ELISA was performed according to the manufacturer’s instructions (BioVendor R&D, Brno, Czech Republic). Both the TGFβ1 and calcitriol investigations were previously described in detail (Lemieszek et al., 2024).

### 2.4 Isolation and purification of nucleic acids and proteins

Unless otherwise indicated, the procedures described below were conducted using reagents and consumables presented in the AllPrep DNA/RNA/Protein Mini Kit (Qiagen, Hilden, Germany). Lung samples were placed in Lysing Matrix M tubes (MP Biomedicals, Solon, United States) in the presence of 600 µL Buffer RLT, and tissues were homogenized mechanically using a FastPrep-24 5G homogenizer (MP Biomedicals) under the following conditions: 6 m/s, 40 s, 20°C. The homogenates were further centrifuged (10,000 × G, 5 min, 4°C), and the collected supernatants were passed through a 70-μm nylon mesh to an AllPrep DNA spin column placed in the collection tube. After centrifugation (8,000 × G, 1 min, 4°C), 250 µL of 96% ethanol was added to the collected filtrate. Then, 600 µL of the sample was transferred to an RNeasy spin column placed in the collection tube. After centrifugation (8,000 × G, 1 min, 4°C), the obtained supernatant was used for further protein extraction, while an RNeasy spin column was used for additional RNA isolation steps.

RNA purification: 700 μL Buffer RW1 was added to the RNeasy spin column, which was centrifuged (8,000 × G, 1 min, 4°C). After discarding the filtrate, 500 μL Buffer RPE was placed in the RNeasy spin column, which was again centrifuged (8,000 x G, 1 min, 4°C). This step was repeated. Next, the RNeasy spin column was transferred to the fresh collection tube. Then, 50 μL RNase-free water was added directly to the spin column, which was centrifuged (8,000 x G, 1 min, 4°C). The amount and quality of eluted RNA were determined using a nanophotometer (Imple, Munich, Germany).

Total protein precipitation: the previously obtained supernatant was mixed with 600 µL Buffer APP and incubated at RT for 10 min on a vortex and then centrifuged (10,600 × G, 10 min, RT). The obtained protein pellet was resuspended in 500 μL of 70% ethanol and centrifuged (10,600 × G, 10 min, RT). The dried protein pellet was resuspended in 150 µL water with 5% SDS. The total protein concentration was determined by a BCA Protein Assay Kit (Thermo Scientific, Rockford, IL, United States).

### 2.5 Evaluation of gene expression: Real-time PCR

RNA (500 ng) was reverse-transcribed using a High Capacity cDNA Reverse Transcription Kit (ThermoFisher Scientific, Vilnius, Lithuania), according to the manufacturer’s instructions. Real-time PCR analysis was carried out using TaqMan Fast Universal PCR MasterMix and TaqMan Gene Expression Assays (Mm00725412_s1 for *Acta2*; Mm02619580_g1 for *Actb*; Mm01247357_m1 for *Cdh1*; Mm01162497_m1 for *Cdh2*; Mm01256744_m1 for *Fn1*; Mm00500912_m1 for *Ocln*; Mm00441533_g1 for *Snail1*; Mm00441531_m1 for *Snail2*; Mm01333430_m1 for *Vim*; Mm00495564_m1 for *Zeb1*; Mm00497196_m1 for *Zeb2*) (Applied Biosystems, Foster City, CA, United States). The reaction was incubated in 96-well optical plates at 95°C for 20 s, followed by 40 cycles of 95°C for 3 s and 60°C for 30 s using a 7,500 Fast Real-Time PCR System (Applied Biosystems, Waltham, MA, United States). Relative expressions were calculated using the efficiency method (relative advanced quantification) and normalized to the expression of *Actb* (SDS 1.4 Software for the 7,500 Fast System, Applied Biosystems, Waltham, MA, United States).

### 2.6 Evaluation of protein expression: Western blotting

The obtained protein samples were solubilized in a Laemmli sample buffer and boiled for 5 min. The samples were adjusted for equal protein loading before being electrophoresed by 8%–14% SDS-PAGE and then transferred to a polyvinylidene difluoride (PVDF) membrane. The membranes were blocked for 1 h at RT with 5% non-fat dry milk in TBS-0.1% Tween 20 (TBS-T) before being probed with primary antibodies directed against β-Actin, E-cadherin, N-cadherin, vimentin (Cell Signaling Technology, Danvers, MA, United States), occludin, α-smooth muscle actin, and fibronectin (Invitrogen, Rockford, United States) at 4°C overnight, followed by incubation with dedicated secondary antibodies conjugated with horseradish peroxidase (Cell Signaling Technology, Danvers, MA, United States) for 1 h at room temperature. Next, the protein expression was visualized on Carestream BioMax Light film (Carestream Health, Rochester, NY, United States) using enhanced chemiluminescence (Thermo Scientific, Rockford, IL, United States).

### 2.7 Examination of signs of inflammation and fibrosis in lung tissue: Masson trichrome staining

Lung tissue samples were fixed and stored in 4% buffered formalin. After dehydration, the lung samples were embedded in paraffin wax. Next, 5-μm-thick sections were obtained from the paraffin blocks and stained with the Masson trichrome method according to the instructions previously presented (Van De Vlekkert et al., 2020). Microscopic preparations were evaluated in an MW 50 light microscope (OPTA-TECH, Warsaw, Poland), while micrographs were prepared in Capture V2.0 software (OPTA-TECH, Warsaw, Poland). The histological examination was performed by a pathologist, who was blinded to the experimental protocol. The fibrosis range was graded with the five-point Murray scale: 0 = regular tissue; 1 = slight injury 25%; 2 = moderate injury 50%; 3 = severe injury 75%; 4 = very severe injury 100%.

### 2.8 Statistical analysis

Statistical analysis of differences between the groups was conducted using the non-parametric Wilcoxon–Mann–Whitney test. To account for multiple comparisons, the Benjamini–Hochberg procedure was applied to control the false discovery rate. A corrected p-value of less than 0.05 was considered statistically significant. The magnitude of the difference between groups was estimated using the Hodges–Lehmann estimator, which provides the median of all pairwise differences between observations in the compared groups. The statistical power of the Wilcoxon–Mann–Whitney test was evaluated using the shiehpow function from the wmwpow R package (version 0.1.3) for estimating the probability of one observation being greater than another (P (X > Y)). Correlations between variables were assessed using Spearman’s rank correlation coefficient. All statistical computations and data visualizations were performed in the R environment (version 4.1.3), utilizing the ggpubr and ggplot2 packages. Research groups consisted of six animals, and the collected lung samples were analyzed twice.

## 3 Results

### 3.1 Inhalation with vitamin D3 metabolites reduced pulmonary fibrosis caused by chronic exposure to antigens of *P. agglomerans*

**FIGURE 2:**
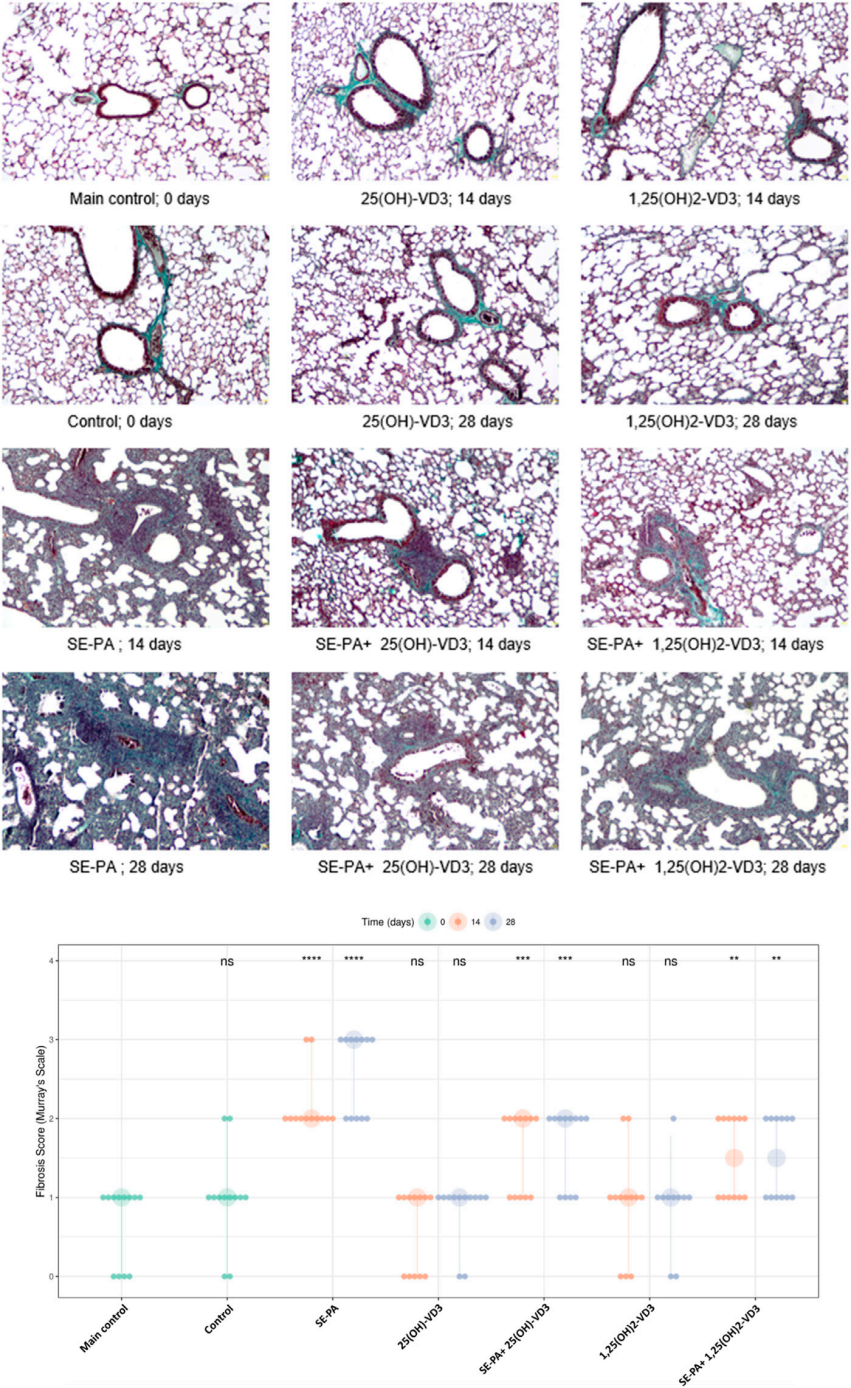
Morphological changes in the lungs of VD3-deficient mice that inhaled vitamin D3 metabolites and the antigen of *Pantoea agglomerans*. Lung tissue samples collected from untreated mice and animals exposed to the assessed compounds were stained according to Masson’s trichrome procedures and investigated under light microscopy. The representative photographs of mouse lung sections stained with Masson’s trichrome. Magnification ×100. Quantification of lung fibrosis scores across various treatment groups and time points. Data are presented with large points representing the median fibrosis score for each condition, while the vertical bars indicate the interquartile range (IQR). The smaller overlaid dots show the scores of individual animals. The time of measurement (in days) is denoted by color, as shown in the legend. Statistical significance was determined using a two-sided Wilcoxon rank-sum test, comparing each treatment group at each time point against the main control group. Asterisks denote the level of statistical significance as follows: *p < 0.05, **p < 0.01, ***p < 0.001, and ****p < 0.0001; ns: not significant. Numerical data are presented in Supplementary Table S2.

As presented in Figure 2, quantification of fibrosis in the murine lungs staining with Masson trichrome did not show any statistically significant changes in these parameters in response to cholecalciferol restriction or nebulization in VD3-deficient mice with investigated metabolites. However, focally thickened alveolar walls were observed in the indicated research groups. On the other hand, VD3-deficient mice that inhaled the antigen of *P. agglomerans* showed fibrosis, the signs of which were distortion and thickening of alveolar walls and emphysema (median score for fibrosis: 2.00) observed after 14 days of treatment. An additional 14 days of exposure to SE-PA accelerated lung destruction, leading to obstruction of the pulmonary alveoli lumen (median score for fibrosis: 3.00). The mentioned increase in fibrosis scores was statistically significant, compared to untreated VD3-sufficient and untreated VD3-deficient mice. VD3 metabolites administered with SE-PA significantly inhibited the profibrotic effect of bacterial antigens; however, focal thickening of the alveolar walls was still recorded. Median scores for fibrosis in response to 25(OH)-VD3 intervention were as follows: 2.00 (SE-PA+ 25(OH)-VD3, 14 days) and 2.00 (SE-PA+ 25(OH)-VD3, 28 days). Median scores for fibrosis recorded after 1,25(OH)_2_-VD3 treatment reached 1.50 in both research groups (SE-PA+ 1,25(OH)2-VD3, 14 days; and SE-PA+ 1,25(OH)2-VD3, 28 days).

**FIGURE 3:**
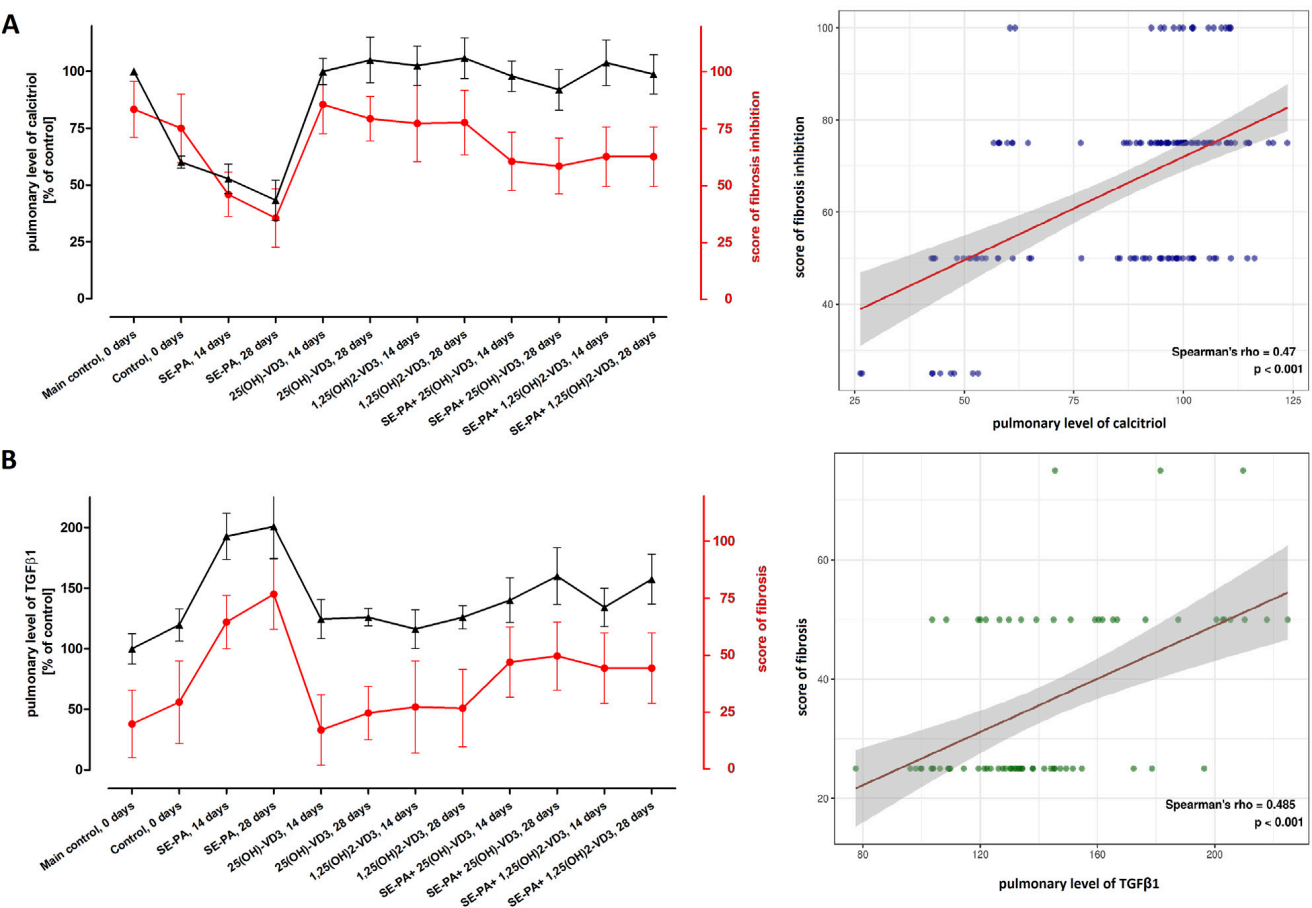
Comparison of longitudinal trends for the pulmonary level of calcitriol with the degree of fibrosis inhibition and amount of TGF-β1. The concentrations of calcitriol and TGFβ1 were determined in lung tissue homogenates using the ELISA method. The results were presented as % of the main control level. Pulmonary fibrosis was scored according to the five-point Murray scale: regular tissue = 0%; slight injury = 25%; moderate injury = 50%; severe injury = 75%; very severe injury = 100%. Scores of fibrosis inhibition were calculated based on fibrosis scores (reversing data), wherein regular tissue was described as 100% fibrosis inhibition, while very severe fibrosis was scored as 0% inhibition. **(A)** The plot displays the relationship between calcitriol (%) and fibrosis inhibition (%). **(B)** The plot shows the relationship between TGFB1 (%) and fibrosis score (%). For both plots, the visualization was made across all research groups and time points. The points on the graph represent the median value of each parameter at the specified time point. These points are connected by lines to illustrate the trends over time. Spearman’s rho and p-value are shown. Numerical data are presented in Supplementary Table S3 and Supplementary Figure S1.

Figure 3 presents the data from the measurement of calcitriol and TGFβ1 concentrations in the mouse lungs and the result of the histopathological evaluation of the lungs in terms of the degree of fibrosis, converted to the percentage of control. The graphical summary of the indicated data revealed that changes in the calcitriol level correspond with the range of pathological changes (the higher the calcitriol level, the stronger the inhibition of fibrosis). Simultaneously, the changes in the TGFβ1 concentration correspond to the degree of fibrosis (the higher the TGFβ1 level, the greater the scale of fibrosis). Comparison of longitudinal trends for paired biomarkers across various research groups revealed positive correlations. The relationship between pulmonary calcitriol level and fibrosis inhibition score was characterized by Spearman’s rho: 0.47 (p < 0.001). Similarly, the relationship between the pulmonary concentration of TGFβ1 and the fibrosis score was characterized by Spearman’s rho: 0.49 (p < 0.001).

### 3.2 Inhalation of vitamin D3 metabolites inhibited the expression of genes involved in EMT in the murine model of HP

**FIGURE 4:**
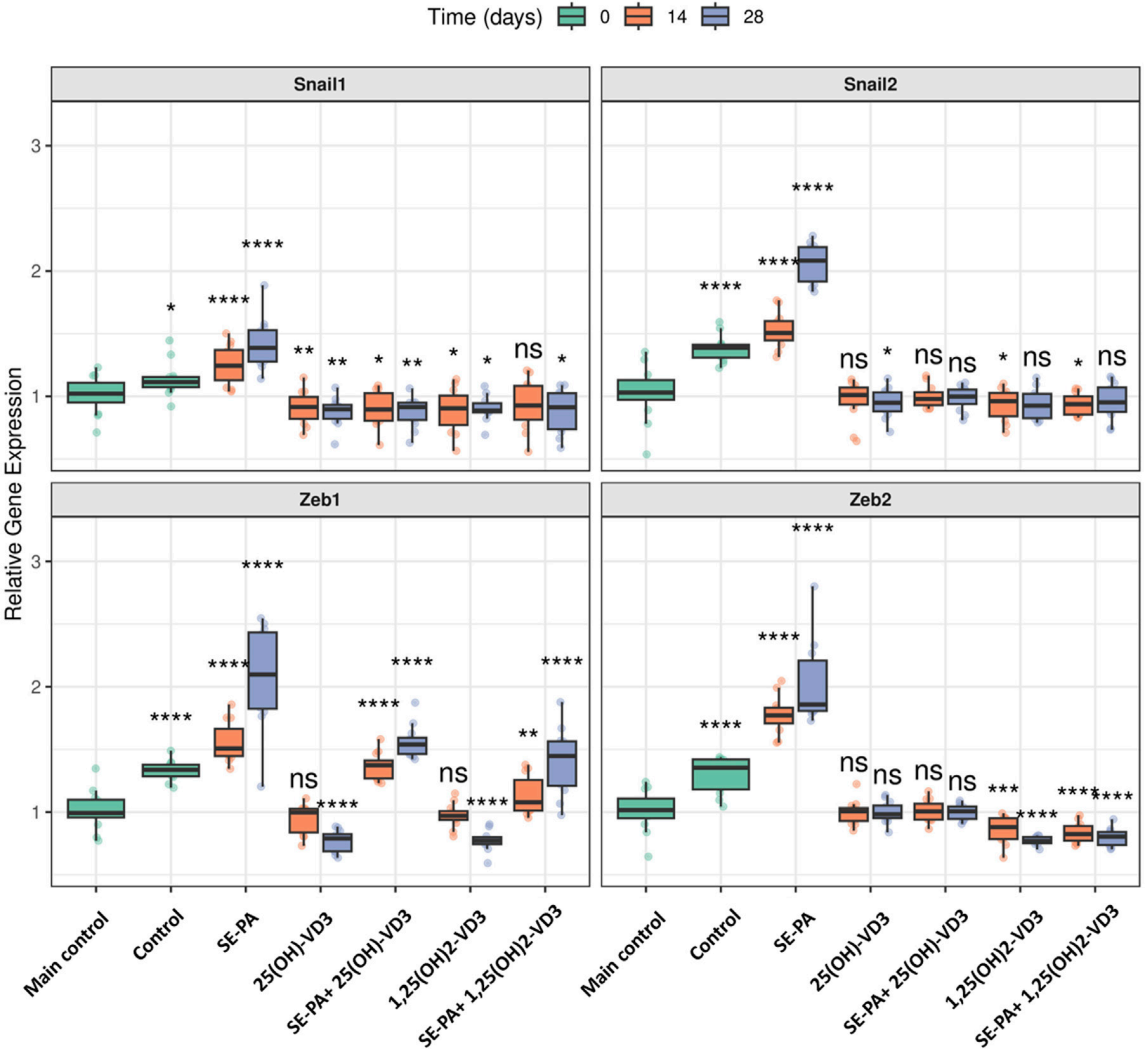
Alterations in the gene expression of transcription factors involved in EMT in the lungs of VD3-deficient mice that inhaled vitamin D3 metabolites and the antigen of *Pantoea agglomerans*. Gene expression was investigated in lung homogenates using real-time PCR methods. Relative gene expression is presented as boxplots where the central line indicates the median, box edges represent the interquartile range (IQR), and whiskers extend to 1.5 times the IQR. Individual data points are overlaid as jittered dots. Data are faceted by gene, with experimental groups shown on the x-axis, and time points (in days) distinguished by color. Statistical significance was determined using the Wilcoxon rank-sum test to compare each treatment group at each time point against the main control group. Significance levels are denoted by asterisks: *p < 0.05, **p < 0.01, ***p < 0.001, and ****p < 0.0001; ns: not significant. Numerical data as well as in-depth statistical analysis are presented in Supplementary Figure S2.

As presented in Figure 4, VD3-deficiencies significantly increased expression of the main EMT transcription factors (*Snail1*: 1.11 vs. 1.02; *Snail2*: 1.39 vs. 1.03; *Zeb1*: 1.34 vs. 0.99; *Zeb2*: 1.35 vs. 1.02).

VD3-deficient mice exposed to vitamin D3 metabolites significantly downregulated the expression of EMT transcription factors. The 14 days of mice exposure to 25(OH)-VD3 restored the physiological level of three of the four tested genes (*Snail2*: 1.01; *Zeb1*: 1.00; *Zeb2*: 1.01), while the long-term experiment indicated a pattern of changes was maintained in the case of one of the four genes (*Zeb2*: 0.98). The 14 days of 1,25(OH)_2_-VD3 nebulization maintained the expression of *Zeb1* (0.97) on the main control level, while a similar pattern of changes was noted in the case of *Snail2* (0.93) after chronic exposure to the tested compound. In other cases, vitamin D3 nebulization decreased the gene expression that had been elevated by VD3 deficiencies to levels less than those of the main control level. The indicated situation was noted in the case of *Snail2* (0.95) and *Zeb1* (0.79) after 28 days of 25(OH)-VD3 exposure, as well as *Snail1* in both investigated time points (0.92 and 0.90). Similarly, 1,25(OH)_2_-VD3 inhalation downregulated the mRNA level of *Snail1* (0.90 and 0.89) and *Zeb2* (0.88 and 0.77) in both tested time points and of *Snail2* (0.96) on the 14th day and *Zeb1* (0.77) on the 28th day of the experiment.

VD3-deficient mice exposed to the antigen of *P. agglomerans* upregulated the gene expression of all factors involved in EMT compared to both the main control and the control. The mRNA levels for the investigated genes reached the following values: (*Snail1*: 1.24 and 1.39; *Snail2*: 1.51 and 2.08; *Zeb1*: 1.51 and 2.10; *Zeb2*: 1.77 and 1.86).

Induced by both SE-PA and VD3-deficiency, the upregulation of EMT transcription factors was effectively reduced by inhalation of vitamin D3 metabolites. Mice exposed to both SE-PA and 25(OH)-VD3 showed the following changes in the tested genes (*Snail1*: 0.90 and 0.91; *Snail2*: 0.98 and 1.00; *Zeb1*: 1.37 and 1.54; *Zeb2*: 1.00 and 1.01). Similarly, animals that inhaled both SE-PA and 1,25(OH)_2_-VD3 showed the following alterations in the evaluated genes (*Snail1*: 0.93 and 0.91; *Snail2*: 0.94 and 0.95; *Zeb1*: 1.08 and 1.45; *Zeb2*: 0.82 and 0.80). The main control level of the indicated genes in VD3-deficient mice with HP was restored after 14 days and 28 days of treatment with 25(OH)-VD3 in the case of *Snail2* and *Zeb2*; 14 days of treatment with 1,25(OH)_2_-VD3 in the case of *Snail1*; 28 days of exposure to 1,25(OH)_2_-VD3 in the case of *Snail2*. It is worth mentioning that the 25(OH)-VD3 administered with SE-PA decreased the expression of *Snail1* to values significantly lower than those detected in the main control at both examined time points, while a similar pattern of changes was recorded in the case of *Snail2* and *Snail1* on the 14th and 28th days of the experiment in the 1,25(OH)_2_-VD3 treatment group.

**FIGURE 5:**
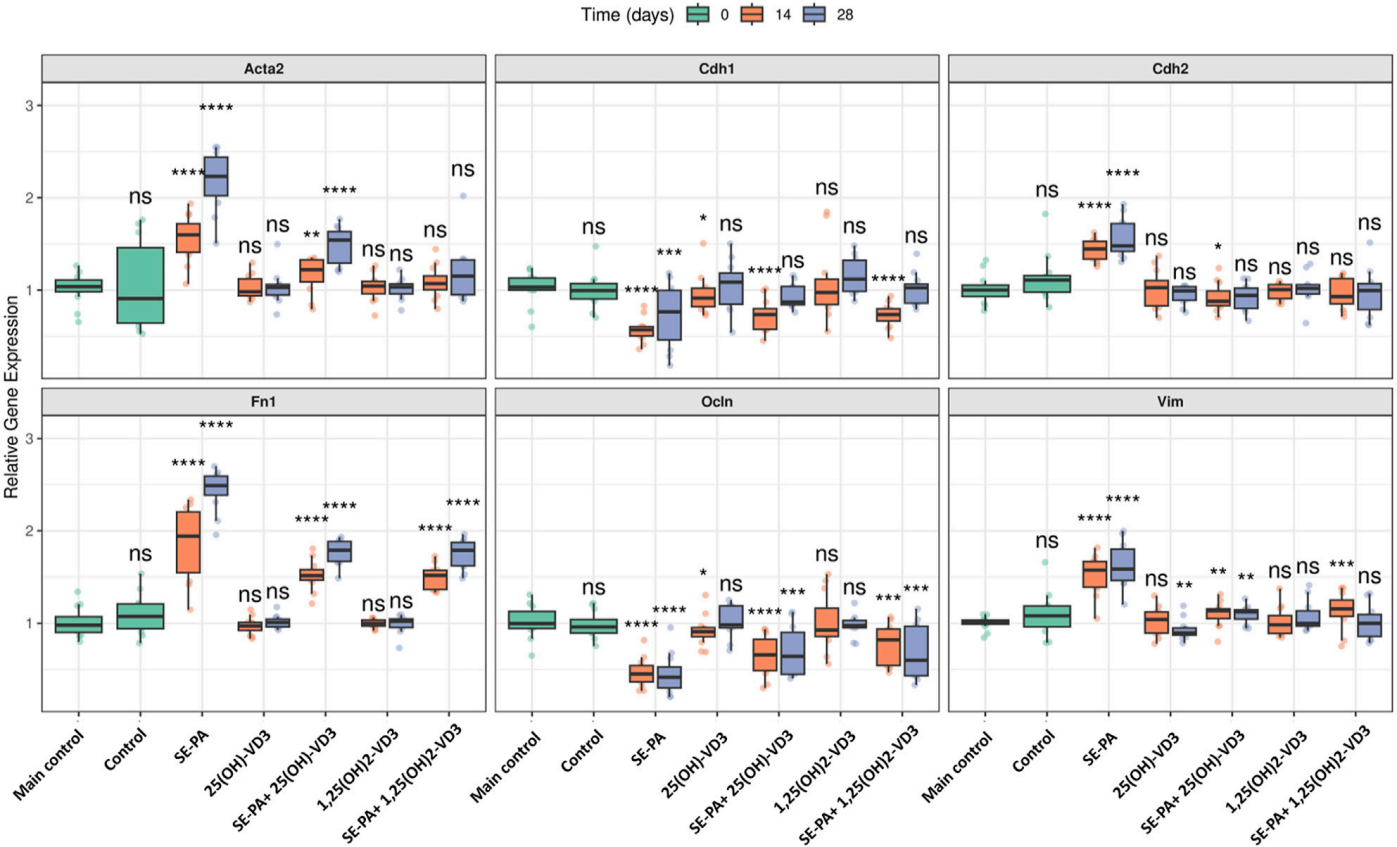
Alterations in the gene expression of epithelial and mesenchymal cell markers in the lungs of VD3-deficient mice that inhaled vitamin D3 metabolites and the antigen of *Pantoea agglomerans.* Gene expression was investigated in lung homogenates using real-time PCR methods. Relative gene expressions are presented as boxplots where the central line indicates the median, box edges represent the interquartile range (IQR), and whiskers extend to 1.5 times the IQR. Individual data points are overlaid as jittered dots. Data are faceted by gene, with experimental groups shown on the x-axis and time points (in days) distinguished by color. Statistical significance was determined using the Wilcoxon rank-sum test to compare each treatment group at each time point against the main control group. Significance levels are denoted by asterisks: *p < 0.05, **p < 0.01, ***p < 0.001, and ****p < 0.0001; ns: not significant. Numerical data as well as in-depth statistical analysis are presented in Supplementary Figure S2.

As presented in Figure 5, the dietary amount of cholecalciferol did not affect the gene expression of both epithelial (*Cdh1, Ocln*) and mesenchymal (*Cdh2, Acta2, Fn1, Vim*) cell markers.

VD3-deficient mice that inhaled 1,25(OH)_2_-VD3 did not show any changes in the expression of the investigated genes. Similarly, the expression of most of the examined genes was also not affected by 25(OH)-VD3 intervention, except the mRNA level of *Cdh1* and *Ocln* noted after 14 days of treatment and *Vim* recorded on the 28th day of the experiment (a significant decrease compared to the control mice was noted; *Cdh1*: 0.92 vs. 1.03; *Ocln*: 0.91 vs. 1.00; *Vim*: 0.90 vs. 1.01).

In contrast, mice exposed to the antigen of *Pantoea agglomerans* altered the mRNA level of EMT markers by downregulating the expression of *Cdh1* and *Ocln* and upregulating the expression of *Cdh2, Acta2, Fn1,* and *Vim*. The expression of mesenchymal cell markers intensified during the treatment with SE-PA (*Acta2*: 1.60 and 2.23; *Fn1*: 1.94 and 2.49), while the expression of other examined markers on the 14th and 28th day of the experiment was quite similar (*Cdh2*: 1.45 and 1.48; *Vim* 1.57 and 1.59; *Cdh1*: 0.57 and 0.77; *Ocln*: 0.45 and 0.42).

Disorders in the expression of EMT markers induced by SE-PA were effectively reduced by mice nebulization with vitamin D3 metabolites. 25(OH)-VD3 administered with SE-PA significantly decreased the expression of mesenchymal cell markers elevated by the antigen. The expressions of mesenchymal markers after 14 days and 28 days of mice inhalation of the antigen of *P. agglomerans* and 25(OH)-VD3 were as follows: *Acta2*: 1.22 and 1.54; *Cdh2*: 0.88 and 0.94; *Fn1*: 1.52 and 1.79; *Vim*: 1.14 and 1.12. It must be highlighted that the mRNA levels of *Cdh2* on the 28th day of the experiment under the indicated conditions reached the main control levels. In the case of epithelial cell markers, only short-term 25(OH)-VD3 treatment effectively inhibited negative changes in the expression of *Cdh1* and *Ocnl* caused by *P. agglomerans* antigen (*Cdh1*: 0.74 vs. 0.57; *Ocln* 14 days: 0.66 vs. 0.45). The expression of *Cdh1* recorded in the indicated research group after 28 days of treatment reached the main control level.

The beneficial impact of vitamin D3 metabolites on the expression of EMT markers, altered by SE-PA, was also observed in response to the bioactive form of vitamin D3. 1,25(OH)_2_-VD3 significantly reduced the level of mesenchymal markers elevated by *P. agglomerans*, the expression of which on the 14th and 28th day of the experiment was as follows: *Acta2*: 1.07 and 1.15; *Cdh2*: 0.93 and 1.00; *Fn1*: 1.52 and 1.79; *Vim*: 1.16 and 1.00. It should be emphasized that the expression of *Cdh2* (both time points), *Acta2* (both time points), and Vim (28th day) under the indicated conditions reached the main control levels. 1,25(OH)_2_-VD3 nebulization also effectively increased the expression of epithelial cell markers downregulated by SE-PA. The mRNA levels of these markers at the investigated time points were as follows: *Cdh1*: 0.74 and 1.03; *Ocln*: 0.82 and 0.60. Expression of *Cdh1* in the long-term experiment in the indicated research group reached the main control level.

### 3.3 Inhalation of vitamin D3 metabolites reversed the negative impact of VD3-deficiencies and antigen *P. agglomerans* exposure on the expression of EMT-related proteins

**FIGURE 6:**
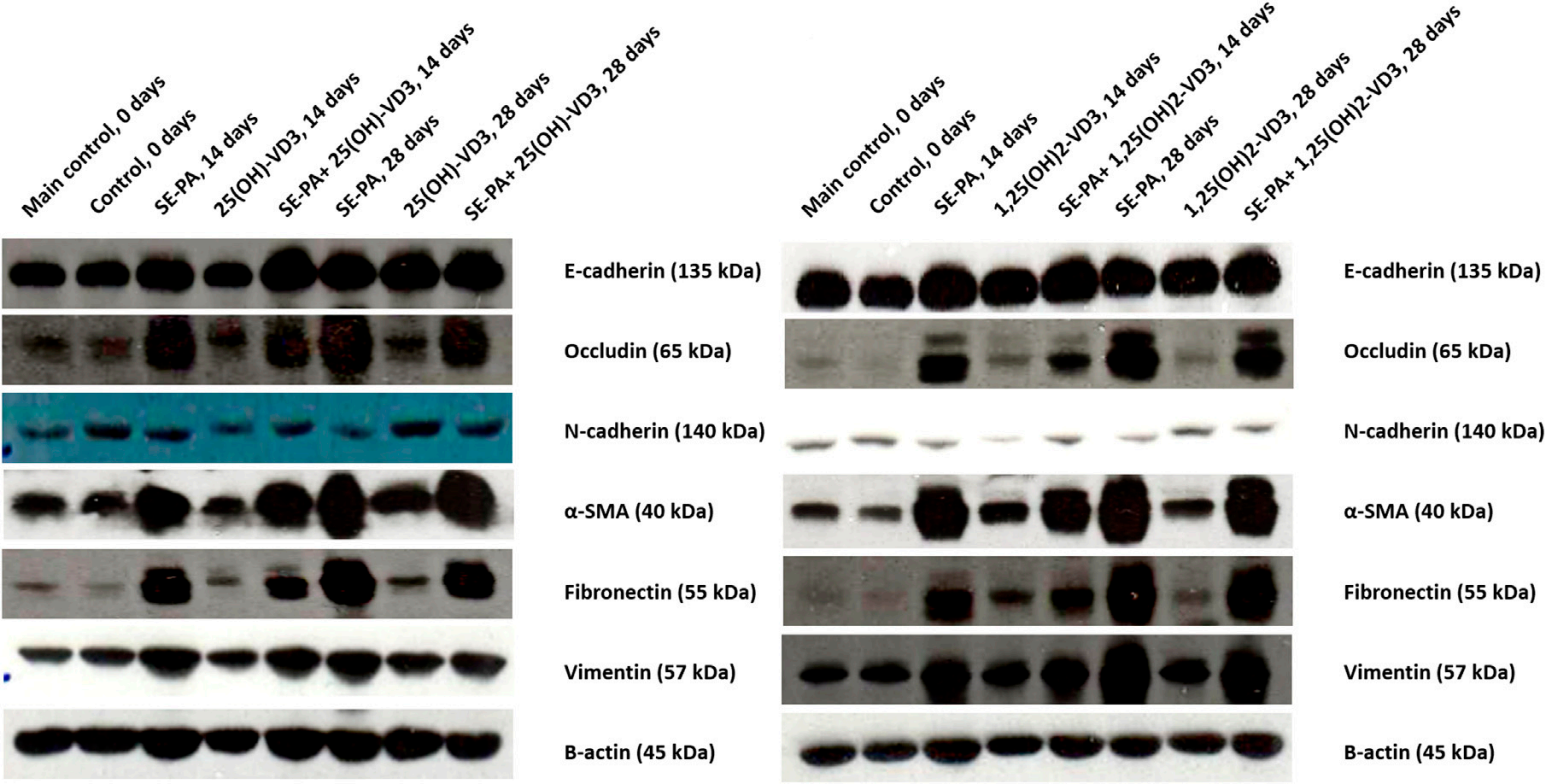
Alterations in the pulmonary expression of epithelial and mesenchymal cell markers in VD3-deficient mice in response to inhalation of vitamin D3 metabolites and the antigen of *Pantoea agglomerans*. Protein expression was investigated in lung homogenates using the Western blotting method. Examination of the ß-actin expression level was used as the internal control. Representative Western blots are shown.

As presented in Figure 6, VD3-deficient mice had a reduced expression of E-cadherin, occludin, α-smooth muscle actin (α-SMA), and fibronectin. At the same time, cholecalciferol deprivation increased the amount of N-cadherin and vimentin.

VD3-deficient mice nebulization with 25(OH)-VD3 and 1,25(OH)_2_-VD3 increased the expression of most of the investigated proteins, compared to the control. A different pattern of changes was recorded in the case of N-cadherin, the amount of which after 14 days of treatment with VD3 metabolites was lower than that recorded in the control, while after 28 days of exposure increased in response to 25(OH)-VD3 or did not change in response to 1,25(OH)_2_-VD3.

VD3-deficient mice chronically exposed to the antigen of *P. agglomerans* significantly increased the expression of occludin, α-SMA, fibronectin, and vimentin and decreased the level of N-cadherin. In contrast, SE-PA did not impact the expression of E-cadherin.

Simultaneous administration of *P. agglomerans* and VD3 metabolites significantly increased the expression of all examined proteins, except N-cadherin, which was lower in the research groups than in untreated mice. Comparison data obtained from HP mice with mice exposed to both VD3 metabolites and SE-PA revealed different patterns of changes. Animals treated with 25(OH)-VD3 and 1,25(OH)_2_-VD3 showed reduced expressions of occludin, fibronectin, and vimentin that had been increased by the antigen. In contrast, the amount of N-cadherin, which was reduced by SE-PA, and the expression of E-cadherin, which was not affected by the antigen, were increased in response to the metabolite treatment. Elevated by SE-PA, the expression of α-SMA did not change after 14 days of treatment with VD3 metabolites. In contrast, 28 days of nebulization with 25(OH)-VD3 increased α-SMA expression, while inhalation of 1,25(OH)_2_-VD3 decreased α-SMA expression.

## 4 Discussion

Our previous study showed that VD3-deficiency disturbed the immune balance, reduced the percentage of macrophages, neutrophils, dendritic cells, and Th lymphocytes in the lungs of mice, and destabilized the level of both proinflammatory and profibrotic cytokines. At the same time, increased concentrations of profibrotic factors (TGFβ, FGF2, hydroxyproline, fibronectin, and type I collagen) were observed in the lungs of VD3-deficient mice. All these together promoted the development of HP in response to the antigen of *P. agglomerans*, which was accompanied by inflammation (granulocyte infiltration, mainly neutrophils) and deformation of lung tissue structure, especially thickening of alveoli walls until complete closing of their lumen, which was noted on the 28th day of SE-PA inhalation. Development of lung fibrosis with prolonged exposure of mice to *P. agglomerans* antigen was associated with progressive intensification of deposition of the extracellular matrix components; increased concentrations of TGFβ and FGF2; disorders of respiratory function, including the increase of frequency of breathing (F) and a decrease of inspiratory and expiratory times (Ti and Te) in both investigated time points, as well as an increase of MV (minute volume) and EF50 (mid-tidal expiratory flow) on day 28 of inhalations.

Pathological changes caused by both VD3-deficiency and exposure to *P. agglomerans* antigen were effectively eliminated by 25(OH)-VD3 and 1,25(OH)_2_-VD3 inhalation, and the beneficial effect of the tested metabolites resulted from their ability to restore physiological serum and pulmonary levels of 1,25(OH)_2_-VD3. However, the pattern of changes caused by 25(OH)-VD3 and 1,25(OH)_2_-VD3 treatment was slightly different depending on the duration of inhalation. The percentage of neutrophils, dendritic cells, and B lymphocytes increased by vitamin D3 deficiency and 14 days of exposure to the antigen of *P. agglomerans* was significantly decreased in response to vitamin D3 metabolites. Similarly, the lowered levels of IL-10, IL-13, and IFN-γ in VD3-deficient mice with HP increased in response to 14 days of 25(OH)-VD3 and 1,25(OH)_2_-VD3 treatment. Moreover, the combined administration of the tested metabolites with SE-PA reduced the upregulated production of TGFβ, FGF2, hydroxyproline, fibronectin, and type I collagen. Additionally, 25(OH)-VD3 and 1,25(OH)_2_-VD3 short-term treatment stabilized respiratory functions disturbed by the progressive development of HP (F and tidal volume (TV)). On the 28th day of the experiment, the beneficial effect of tested metabolites on the development of lung fibrosis in VD3-deficient mice was primarily associated with the inhibition of the influx of macrophages, neutrophils, Th and Tc lymphocytes (both 25(OH)-VD3 and 1,25(OH)_2_-VD3), as well as dendritic cells and B lymphocytes (1,25(OH)_2_-VD3). In the indicated research groups, the antifibrotic effect of 25(OH)-VD3 and 1,25(OH)_2_-VD3 was also associated with the inhibition of excessive deposition of fibronectin and hydroxyproline, the limitation of TGFβ and FGF2 production, and the stabilization of respiratory parameters, in particular F, MV, EF50 Ti, and Te. The studies have clearly shown that 25(OH)-VD3 and 1,25(OH)_2_-VD3 administered in doses restoring the physiological calcitriol level in the lungs of VD3-deficient mice with HP inhibited the development of lung fibrosis by restoring the immunological balance, in particular by limiting the excessive influx of immune cells into the lung parenchyma and reducing the overproduction of factors promoting inflammation and pathological tissue remodeling (Lemieszek et al., 2024).

Our earlier studies revealed the high antifibrotic potential of 25(OH)-VD3 and 1,25(OH)_2_-VD3 nebulization based on modulation of immune response and deposition of extracellular matrix components, both of which are important components of EMT type 2. The present study focused directly on this phenomenon to better understand the therapeutic effect of vitamin D3 metabolites. To properly understand the results, it should be noted that both VD3-metabolites were used in concentrations required to restore the physiological level of calcitriol disturbed by a VD3-deficient diet, together with chronic exposure to an HP-inducer (antigen of *P. agglomerans*). VD3-metabolite doses were selected based on the results of our earlier studies (Chojnacki et al., 2023; Lemieszek et al., 2024). This study developed and further verified our previously presented concept that the basis of safe and effective therapy for pulmonary fibrosis in the course of HP is the neutralization of 1,25(OH)2-VD3 deficiency in the lung tissue by direct administration of its precursor or exogenous form to the respiratory tract in an amount necessary to restore its physiological concentration (Chojnacki et al., 2023; Lemieszek et al., 2024).

In the beginning, we returned to histological evaluation, but this time, the investigation was performed after Masson trichrome staining, which highlights collagen fibers. Light microscopy observation demonstrated discrete changes in the morphology of pulmonary tissues in VD3-deficient mice that were untreated and in mice exposed to 25(OH)-VD3 or 1,25(OH)_2_-VD3. The local thickening of the alveoli walls was noted. In contrast, VD3-deficient mice exposed to profibrotic *P. agglomerans* antigen accelerated collagen deposition in a time-dependent manner, leading to complete closure of the alveoli lumen on day 28 of the experiment. Simultaneously, both 25(OH)-VD3 and 1,25(OH)_2_-VD3 treatment reduced the negative impact of both SE-PA and VD3 deficiencies on wound healing, as evidenced by the replacement of lung parenchyma by extracellular matrix components. A comparison of the fibrosis inhibition scores with the pulmonary level of calcitriol showed an inverse relationship between the concentration of the tested metabolite and the range of pathological changes. This observation proves the postulate of the existence of a connection between the development of pulmonary fibrosis in the course of HP and the pulmonary level of calcitriol (Chojnacki et al., 2023; Chojnacki and Lemieszek, 2023; Lemieszek et al., 2024). These comparisons confirm the main assumption of our concept of HP prevention by maintaining or restoring the physiological pulmonary level of calcitriol using inhalation with vitamin D3 metabolites (Chojnacki et al., 2023; Chojnacki and Lemieszek, 2023; Lemieszek et al., 2024). Furthermore, the results of our earlier studies (Lemieszek et al., 2024), a graphical representation of which is presented in the current manuscript, revealed that the range of pulmonary fibrosis correlated with the pulmonary level of TGFβ1—the best-known and best-described EMT inducer. The indicated data suggest that the modulation of the EMT process by vitamin D3 metabolites may be responsible for their beneficial effect on the course of lung fibrosis in HP and consequently inspired us in this study. Thus, to understand the molecular mechanism of the discovered antifibrotic action of vitamin D3 metabolites, the expression of EMT components was examined on both the mRNA and protein levels.

Real-time PCR focused on the key EMT player has shown that cholecalciferol diet restriction significantly increased the expression of EMT transcription factors (*Snail1, Snail2, Zeb1,* and *Zeb2*) but did not affect the expression of epithelial and mesenchymal markers (*Acta2, Cdh1, Cdh2, Fn1, Ocln,* and *Vim*). VD3-deficient mice that inhaled 25(OH)-VD3 and 1,25(OH)_2_-VD3 effectively reduced the indicated overexpression of EMT factors, which, in our opinion, was associated with restoration of the physiological level of calcitriol in lung tissue.

Similarly, a drastic decline in the pulmonary level of calcitriol in VD3-deficient mice exposed to SE-PA corresponded with the downregulation of epithelial cell markers (*Cdh1, Ocln*), and simultaneously, upregulation of mesenchymal cell markers (*Acta2, Cdh2, Fn1, Vim*) and overexpression of EMT transcription factors (*Snail1, Snail2, Zeb1, Zeb2*). Indicated changes in the gene expression intensified with the duration of the antigen exposure and decreasing pulmonary concentration of calcitriol.

Our earlier study conducted on VD3-sufficient mice with HP (Lemieszek et al., 2020; Lemieszek et al., 2022) revealed a similar expression profile of genes encoding key EMT factors; however, comparison of previous and current data demonstrated significant differences in mRNA levels, dependent on the amount of cholecalciferol in the animals’ diet. Compared to VD3-sufficient mice exposed to the same dose of SE-PA for the same period, VD3 deficiency increased the expression of mesenchymal cell markers and EMT transcription factors and reduced the level of epithelial cell markers. Indicated differences in the expression of EMT genes recorded in murine models of HP could be caused not only by VD3 status but also by animal gender. Our earlier studies were conducted on female mice, while the current data were collected from male mice.

The gene expression profile reported in VD3-deficient mice exposed to SE-PA was quite similar to the changes observed in other pulmonary fibrosis models, including the murine model of IPF (sameness was observed in the cases of *Acta2, Fn1*) (Tzilas et al., 2019; Chang et al., 2021), as well as human bronchial epithelial cells exposed to TGFβ (sameness was noted in the cases of *Acta2, Cdh1, Cdh2, Vim,* and *Snail*) (Fischer and Agrawal, 2014; Zheng et al., 2020). EMT induction recorded on the mRNA level in reported research was inhibited by animal supplementation with cholecalciferol (Tzilas et al., 2019) or the bioactive analog of vitamin D3, paricalcitol (Chang et al., 2021), while beneficial changes in pulmonary epithelium were associated with cell incubation with 25(OH)-VD3 (Zheng et al., 2020) and 1,25(OH)_2_-VD3 (Fischer and Agrawal, 2014; Zheng et al., 2020). Our investigation also proved the therapeutic potential of 25(OH)-VD3 and 1,25(OH)_2_-VD3, based on restoring balance in the expression of EMT-related genes disturbed by SE-PA exposure and cholecalciferol dietary restriction. The studies demonstrated that VD3-deficient mice with HP that inhaled the tested VD3 metabolites increased over-reduced expression of *Cdh1* and *Ocl* and simultaneously decreased over-elevated expression of *Acta2, Cdh2, Fn1, Vim, Snail1, Snail2, Zeb1,* and *Zeb2*. Full recovery in response to 25(OH)-VD3 treatment was observed in the case of Snail2 and Zeb2 (both time points) and in Cdh1 and Cdh2 (28 days of inhalations). In contrast, restoration of physiological gene expression levels in response to 1,25(OH)2-VD3 treatment was recorded in the case of Acta2 and Cdh2 (both time points), Snail1 (14 days of inhalations), Cdh1, Snail2, and Vim (28 days of inhalations). The beneficial influence of 25(OH)-VD3 and 1,25(OH)_2_-VD3 nebulization on EMT induction in VD3-deficient mice with HP seems to be associated with the restoration of the physiological pulmonary level of calcitriol.

Despite the results of gene expression assays indicating that the antifibrotic effect of nebulization with 25(OH)-VD3 and 1,25(OH)_2_-VD3 is based on inhibition of EMT triggered in VD3-deficient mice by SE-PA, further examination conducted on the protein level was unable to draw such a clear conclusion. Several discrepancies in the expression levels of genes and corresponding proteins were noted.

In contrast to real-time PCR data, Western blot showed alterations in the expression of all EMT markers in response to cholecalciferol deprivation. VD3 deficiencies reduced the expression of epithelial markers (E-cadherin and occludin) and mesenchymal markers (α-smooth muscle actin, and fibronectin) and simultaneously increased the amount of vimentin. Differences in transcriptome and proteome levels were also noted in VD3-deficient mice exposed to 25(OH)-VD3 and 1,25(OH)_2_-VD3. Western blot revealed the upregulation of most examined EMT markers except N-cadherin, the expression of which decreased in response to 14 days of inhalation of the tested metabolites. A comparison of results obtained from untreated VD3-deficient mice and from the animals in which inhalation of VD3 metabolites restored the physiological calcitriol level revealed an interesting dependency: inhalation of VD3 metabolites attenuated most of the disorders in the protein expression caused by cholecalciferol deprivation, excluding vimentin (both time points) and N-cadherin (28th day of the experiment).

Although real-time PCR demonstrated the typical pattern of changes for EMT in mice response to SE-PA, Western blots confirmed only accelerated expression of α-smooth muscle actin, fibronectin, and vimentin. In contrast, the expression of E-cadherin was not affected, the amount of occludin was elevated, and the expression of N-cadherin was downregulated. The expression profiles of EMT markers presented here, compared to our earlier research conducted in VD3-sufficient mice with HP, are similar only in the case of α-smooth muscle actin, fibronectin, and vimentin, while they differ in the case of E-cadherin, N-cadherin, and occludin (Lemieszek et al., 2020; Lemieszek et al., 2022). Nevertheless, Fei et al., based on the increased amount of just two mesenchymal cell markers (vimentin and α-SMA) in patients with both chronic obstructive pulmonary disease and VD3-deficiencies, correlated recorded changes with EMT; in contrast to our data, they also observed lowered expression of E-cadherin (Fei et al., 2019). The cited study is not the only one in which pulmonary fibrosis was characterized by increased levels of α-smooth muscle actin, fibronectin, and vimentin (one or two of them) without assessment of other EMT markers. In this light, other data should be mentioned that were collected from murine pulmonary fibroblast stimulated with TGFβ (Ramirez et al., 2010), human pulmonary fibroblast cell lines HPF and HFL1 (Zhu et al., 2021; Sobczak and Pawliczak, 2022), a murine model of IPF (Chang et al., 2021; Zhu et al., 2021), and a rat model of allergic asthma (Huang et al., 2019). Taking the above data into account, the fibrosis observed in VD3-deficient mice chronically exposed to SE-PA may be associated with the induction of EMT.

In the last step of the study, the impact of 25(OH)-VD3 and 1,25(OH)_2_-VD3 inhalation on the expression of EMT molecules in HP mice with VD3 deficiencies was examined. Although the beneficial influence of metabolites on the investigated phenomenon was not as obvious as that observed on the mRNA level, the therapeutic potential of the tested compounds was still noticeable. In the case of epithelial markers, both 25(OH)-VD3 and 1,25(OH)_2_-VD3 treatment accelerated the expression of E-cadherin, as well as inhibiting overexpressed occludin. In the case of mesenchymal cell markers, both 25(OH)-VD3 and 1,25(OH)_2_-VD3 treatment reduced overexpressed fibronectin and vimentin; they simultaneously restored the physiological level of N-cadherin.

Enhancement of epithelial cell markers expression, together with the silencing of heightened expression of mesenchymal cell markers recorded in VD3-deficient mice with HP in response to inhalations with 25(OH)-VD3 and 1,25(OH)_2_-VD3, suggested that the antifibrotic effect of these compounds is associated with inhibition of the EMT phenomenon. The described molecular mechanism of the antifibrotic properties of VD3 metabolites corresponded with data collected from both *in vitro* and *in vivo* models of pulmonary fibrosis. Upregulation of E-cadherin in response to 1,25(OH)_2_-VD3 was observed in human bronchial epithelial BEAS-2B cells stimulated with TGFβ (Fischer and Agrawal, 2014) and rat alveolar epithelial cells stimulated with TGFβ (Xiong et al., 2020). Moreover, 25(OH)-VD3 and 1,25(OH)_2_-VD3 increased the amount of E-cadherin in human type 2 pneumocytes stimulated with TGFβ (Zheng et al., 2020). In contrast, downregulation of vimentin in response to 1,25(OH)_2_-VD3 was observed in several human cell lines stimulated with TGFβ, including type 2 pneumocytes (Zheng et al., 2020), bronchial epithelial BEAS-2B cells (Fischer and Agrawal, 2014), and pulmonary fibroblast HFL1 cells (Sobczak and Pawliczak, 2022), while downregulation of fibronectin in response to 1,25(OH)_2_-VD3 was observed in rodent cell lines treated with TGFβ, including murine pulmonary fibroblasts (Ramirez et al., 2010) and rat alveolar epithelial cells (Xiong et al., 2020). The beneficial impact of 25(OH)-VD3 on vimentin level overexpressed by TGFβ was recorded in human type 2 pneumocytes (Zheng et al., 2020). The therapeutic effect of vitamin D3, based on the reduction of vimentin expression together with an increase in E-cadherin synthesis, was also noted in the murine model of IPF (Chang et al., 2021).

Although the cited antifibrotic effects of vitamin D3 metabolites based on inhibition of EMT mostly came from *in vitro* studies, some *in vivo* research (apart from our latest study, Lemieszek et al., 2024) has investigated the influence of vitamin D3 metabolites administered orally (Huang et al., 2019; Tzilas et al., 2019), by intragastric injection (Zheng et al., 2020), or by intraperitoneal injection (Tan et al., 2016; Chang et al., 2021; Zhu et al., 2021). The presented study is unique from three aspects: 1) the route of administration—direct administration to the respiratory tract via inhalation; 2) as a research model—it is the first study describing the effect of vitamin D3 metabolites on the EMT in the development of pulmonary fibrosis in the course of HP; 3) the concept of the study—based on the direct delivery of vitamin D3 metabolites to the respiratory tract in the amount necessary to restore the physiological level of calcitriol in the pulmonary compartment and prevent the fibrosis development (Chojnacki et al., 2023; Lemieszek et al., 2024).

## 5 Conclusion

In summary, the presented research demonstrated the connection between pulmonary calcitriol level and the range of fibrosis in the murine model of HP. Moreover, the performed study revealed that VD3-deficiency triggers EMT, the signs of which were increased expression of genes coding EMT transcription factors, inhibited expression of epithelial cell markers, and altered expression of mesenchymal cell markers, including upregulated N-cadherin and vimentin. Pathological changes caused by VD3-deficiencies accelerated the murine response to *P. agglomerans* antigen (SE-PA; etiological factor of HP and proven pulmonary fibrosis inducer) in both transcriptome and proteome levels; moreover, the changes were clearly manifested in histological examination. Further studies on the transcriptome and proteome levels confirmed the previously reported antifibrotic potential of inhalation of vitamin D3 metabolites associated with restoring the balance in the expression of EMT molecules disturbed by cholecalciferol deficiency and SE-PA exposure. However, the tested compounds could not wholly prevent the epithelial-to-mesenchymal transition and, consequently, could not completely prevent the development of pulmonary fibrosis. The inhalation of 25(OH)-VD3 and 1,25(OH)_2_-VD3 had a beneficial influence on the HP course, albeit without full recovery of the tested animals, as reflected in histological examination. The presented research is the first to show the influence of VD3 status on EMT in light of the development of fibrosis in the course of HP. The obtained data bring hope for the creation of effective and safe HP therapy based on the restoration of the physiological level of calcitriol in the pulmonary areas.

## Data Availability

The original contributions presented in the study are publicly available. This data can be found here: Zenodo repository, doi: 10.5281/zenodo.15728801.
