# Supplementary material for: The first step for understanding the molecular mechanism of the antifibrotic effect of inhaling 25(OH)-vitamin D3 and 1,25(OH)2-vitamin D3 in the murine model of hypersensitivity pneumonitis
Source: Front Pharmacol. 2025 Aug 8;16:1610165. doi: 10.3389/fphar.2025.1610165 (PMC12370658; doi:10.3389/fphar.2025.1610165)
Supplement: Supplementary file 1 [file Supplementaryfile1.docx]

**Table S1.** Description of research groups

| **Research group**  **(number of animals)** | **Vitamin D3 content in the diet** | **Calcidiol;**  **25(OH)-VD3**  **(100 pg/g)** | **Calcitriol;**  **1,25(OH)_2_-VD3**  **(5 pg/g)** | **SE-PA;**  **saline extract of *Pantoea agglomerans***  **(5 mg/mouse)** |
| --- | --- | --- | --- | --- |
| **Main control 0 days**  **(n = 6)** | **0.5 IU/g** | - | - | - |
| **Control 0 days**  **(n = 6)** | **0.05 IU/g** | - | - | - |
| **SE-PA**  **14 days**  **(n=6)** | **0.05 IU/g** | - | - | 30 minutes of daily inhalation for 14 consecutive days |
| **SE-PA; 28 days**  **(n=6)** | **0.05 IU/g** | - | - | 30 minutes of daily inhalation for 28 consecutive days |
| **25(OH)-VD3**  **14 days**  **(n=6)** | **0.05 IU/g** | 30 minutes of daily inhalation for 14 consecutive days | - | - |
| **25(OH)-VD3**  **28 days**  **(n=6)** | **0.05 IU/g** | 30 minutes of daily inhalation for 28 consecutive days | - | - |
| **1,25(OH)2-VD3**  **14 days**  **(n=6)** | **0.05 IU/g** | - | 30 minutes of daily inhalation for 14 consecutive days | - |
| **1,25(OH)2-VD3**  **28 days**  **(n=6)** | **0.05 IU/g** | - | 30 minutes of daily inhalation for 28 consecutive days | - |
| **SE-PA + 25(OH)-VD3**  **14 days**  **(n=6)** | **0.05 IU/g** | 30 minutes of daily inhalation for 14 consecutive days | - | 30 minutes of daily inhalation for 14 consecutive days |
| **SE-PA + 25(OH)-VD3**  **28 days**  **(n=6)** | **0.05 IU/g** | 30 minutes of daily inhalation for 28 consecutive days | - | 30 minutes of daily inhalation for 28 consecutive days |
| **SE-PA + 1,25(OH)2-VD3**  **14 days**  **(n=6)** | **0.05 IU/g** | - | 30 minutes of daily inhalation for 14 consecutive days | 30 minutes of daily inhalation for 14 consecutive days |
| **SE-PA + 1,25(OH)2-VD3**  **28 days (n=6)** | **0.05 IU/g** | - | 30 minutes of daily inhalation for 28 consecutive days | 30 minutes of daily inhalation for 28 consecutive days |

**Table S2.** Quantification of fibrosis in murine lung tissue in response to inhalation with the antigen of *Pantoea agglomerans* and/or vitamin D3 metabolites. These features were graded with 5-point Murray’s scale: 0 = regular tissue; 1 = slight injury 25%; 2 = moderate injury 50%; 3 = severe injury 75%; 4 = very severe injury 100%. Data for histologic scores are given as median of investigated items. Statistical significance was determined using a two-sided Wilcoxon Rank Sum test, comparing each treatment group at each time point against the Main Control 0 days, Control 0 days, SE-PA 14 days and SE-PA 28 days. Asterisks denote the level of statistical significance as follows: * p < 0.05, ** p < 0.01, *** p < 0.001, and **** p < 0.0001; ns, not significant.

|  | **Main control**  **0 days** | **Control**  **0 days** | **SE-PA**  **14 days** | **SE-PA**  **28 days** | **25(OH)-VD3**  **14 days** | **25(OH)-VD3**  **28 days** | **1,25(OH)2-VD3**  **14 days** | **1,25(OH)2-VD3**  **28 days** | **SE-PA+**  **25(OH)-VD3**  **14 days** | **SE-PA+**  **25(OH)-VD3**  **28 days** | **SE-PA+**  **1,25(OH)2-VD3**  **14 days** | **SE-PA+**  **1,25(OH)2-VD3**  **28 days** |
| --- | --- | --- | --- | --- | --- | --- | --- | --- | --- | --- | --- | --- |
| **Fibrosis score (median)** | 1 | 1 | 2 | 3 | 1 | 1 | 1 | 1 | 2 | 2 | 1,5 | 1,5 |
| **p value vs. Main control 0 days** | - | 0,17490 | 0,00001 | 0,00002 | 0,70516 | 0,37643 | 0,36221 | 0,35209 | 0,00098 | 0,00045 | 0,00194 | 0,00194 |
| **p value vs. Control 0 days** | 0,17490 | - | 0,00011 | 0,00006 | 0,09821 | 0,46906 | 0,76318 | 0,71816 | 0,02495 | 0,01095 | 0,05153 | 0,05153 |
| **p value vs. SE-PA 14 days** | 0,00001 | 0,00011 | - | 0,04242 | 0,00001 | 0,00001 | 0,00012 | 0,00009 | 0,00824 | 0,01637 | 0,00389 | 0,00389 |
| **p value vs. SE-PA 28 days** | 0,00002 | 0,00006 | 0,04242 | - | 0,00002 | 0,00001 | 0,00007 | 0,00009 | 0,00068 | 0,00098 | 0,00045 | 0,00045 |

**Table S3.** Comparison of the pulmonary level of calcitriol with the degree of fibrosis inhibition and amount of TGFβ1. The concentrations of calcitriol as well as TGFβ1 were determined in lung tissue homogenates using the ELISA method. The obtained results were presented as % of the Main control level. Pulmonary fibrosis was scored according to the five-point Murray scale: regular tissue = 0%; slight injury = 25%; moderate injury = 50%; severe injury = 75%; very severe injury = 100%. Scores of fibrosis inhibition were calculated based on fibrosis scores (revering data), wherein regular tissue was described as 100% fibrosis inhibition, while very severe fibrosis was scored as 0% inhibition. Data are presented as median of investigated parameters.

|  | **Main control**  **0 days** | **Control**  **0 days** | **SE-PA**  **14 days** | **SE-PA**  **28 days** | **25(OH)-VD3**  **14 days** | **25(OH)-VD3**  **28 days** | **1,25(OH)2-VD3**  **14 days** | **1,25(OH)2-VD3**  **28 days** | **SE-PA+**  **25(OH)-VD3**  **14 days** | **SE-PA+**  **25(OH)-VD3**  **28 days** | **SE-PA+**  **1,25(OH)2-VD3**  **14 days** | **SE-PA+**  **1,25(OH)2-VD3**  **28 days** |
| --- | --- | --- | --- | --- | --- | --- | --- | --- | --- | --- | --- | --- |
| **Pulmonary level of calcitriol**  **(median)** | 99,67 | 60,08 | 52,23 | 97,11 | 100,51 | 106,48 | 103,30 | 94,77 | 105,81 | 43,90 | 107,44 | 98,38 |
| **Score of fibrosis inhibition  (median)** | 75,00 | 75,00 | 50,00 | 50,00 | 75,00 | 62,50 | 75,00 | 50,00 | 75,00 | 25,00 | 75,00 | 62,50 |
| **Pulmonary level of TGFβ1  (median)** | 103,56 | 117,91 | 201,63 | 207,78 | 133,55 | 125,03 | 117,43 | 129,78 | 145,74 | 164,19 | 138,30 | 162,87 |
| **Fibrosis score**  **(median)** | 25,00 | 25,00 | 50,00 | 50,00 | 25,00 | 37,50 | 25,00 | 50,00 | 25,00 | 75,00 | 25,00 | 37,50 |

| **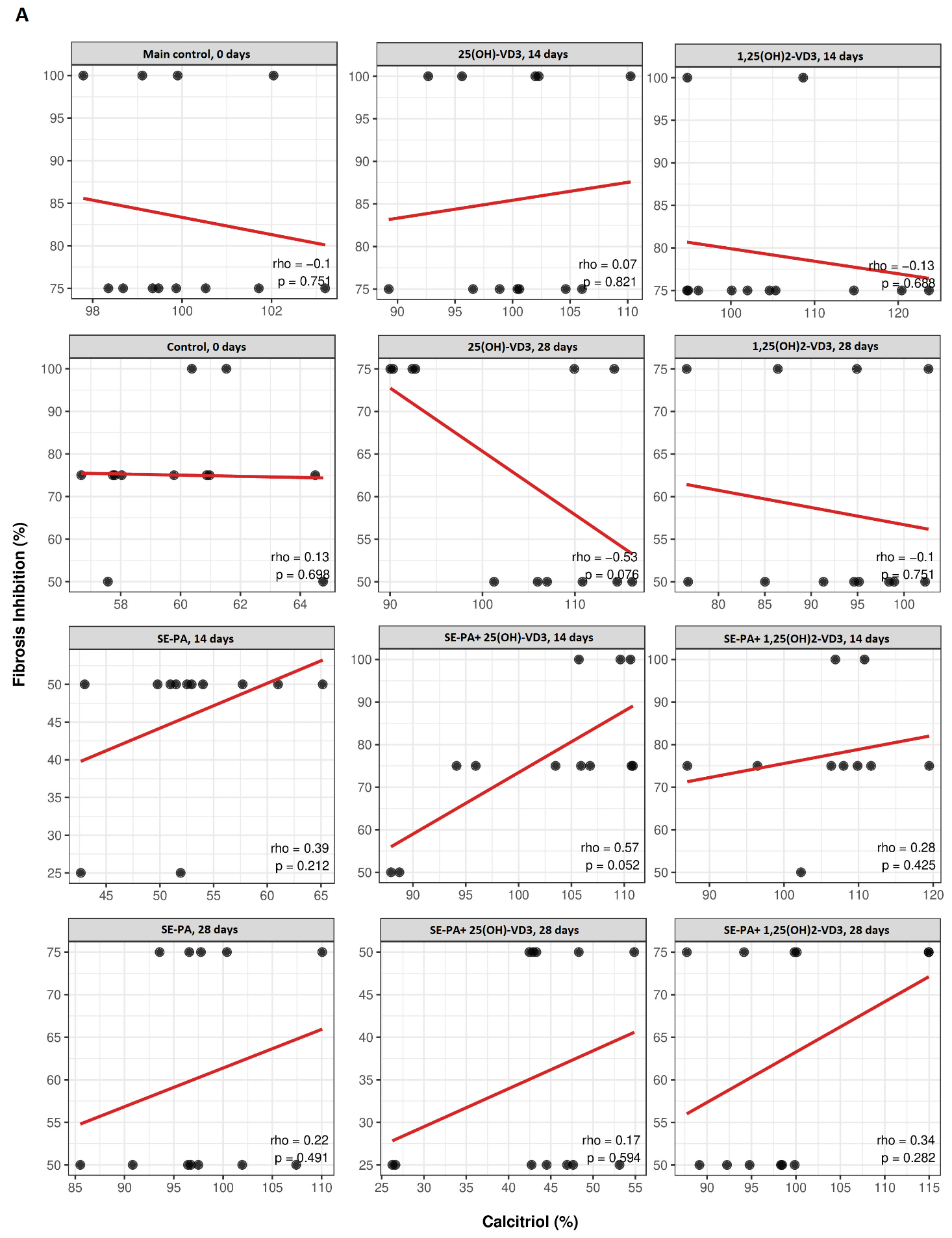** |
| --- |
| **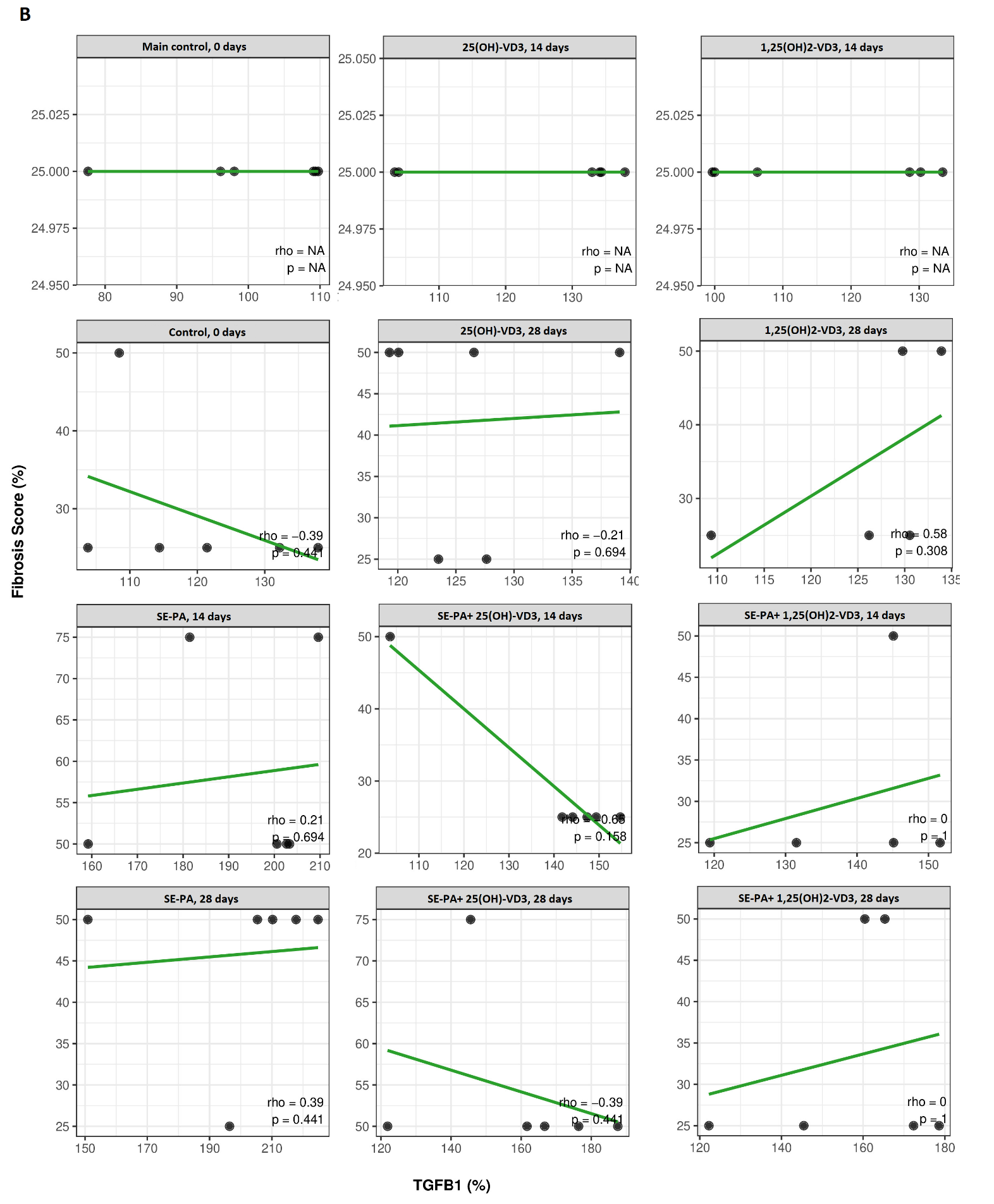** |
| **Figure S1.** Comparison of longitudinal trends for pulmonary level of calcitriol with the degree of fibrosis inhibition and amount of TGFβ1. The concentrations of calcitriol and TGFβ1 were determined in lung tissue homogenates using the ELISA method. The obtained results were presented as % of the Main control level. Pulmonary fibrosis was scored according to the five-point Murray scale: regular tissue = 0%; slight injury = 25%; moderate injury = 50%; severe injury = 75%; very severe injury = 100%. Scores of fibrosis inhibition were calculated based on fibrosis scores (revering data), wherein regular tissue was described as 100% fibrosis inhibition, while very severe fibrosis was scored as 0% inhibition. A) The plot displays the relationship between Calcitriol (%) and Fibrosis Inhibition (%). (B) The plot shows the relationship between TGFB1 (%) and Fibrosis Score (%). For both plots, the visualization is faceted into multiple panels, where each panel corresponds to a specific treatment group. The x-axis within each panel indicates the time in days. A dual y-axis structure is employed to display the two parameters simultaneously; the left axis corresponds to the first parameter mentioned (solid line), and the right axis corresponds to the second (dashed line). The points on the graph represent the median value of each parameter at the specified time points. These points are connected by lines to illustrate the trends over time. Spearman’s rho and p-value are shown. |

| 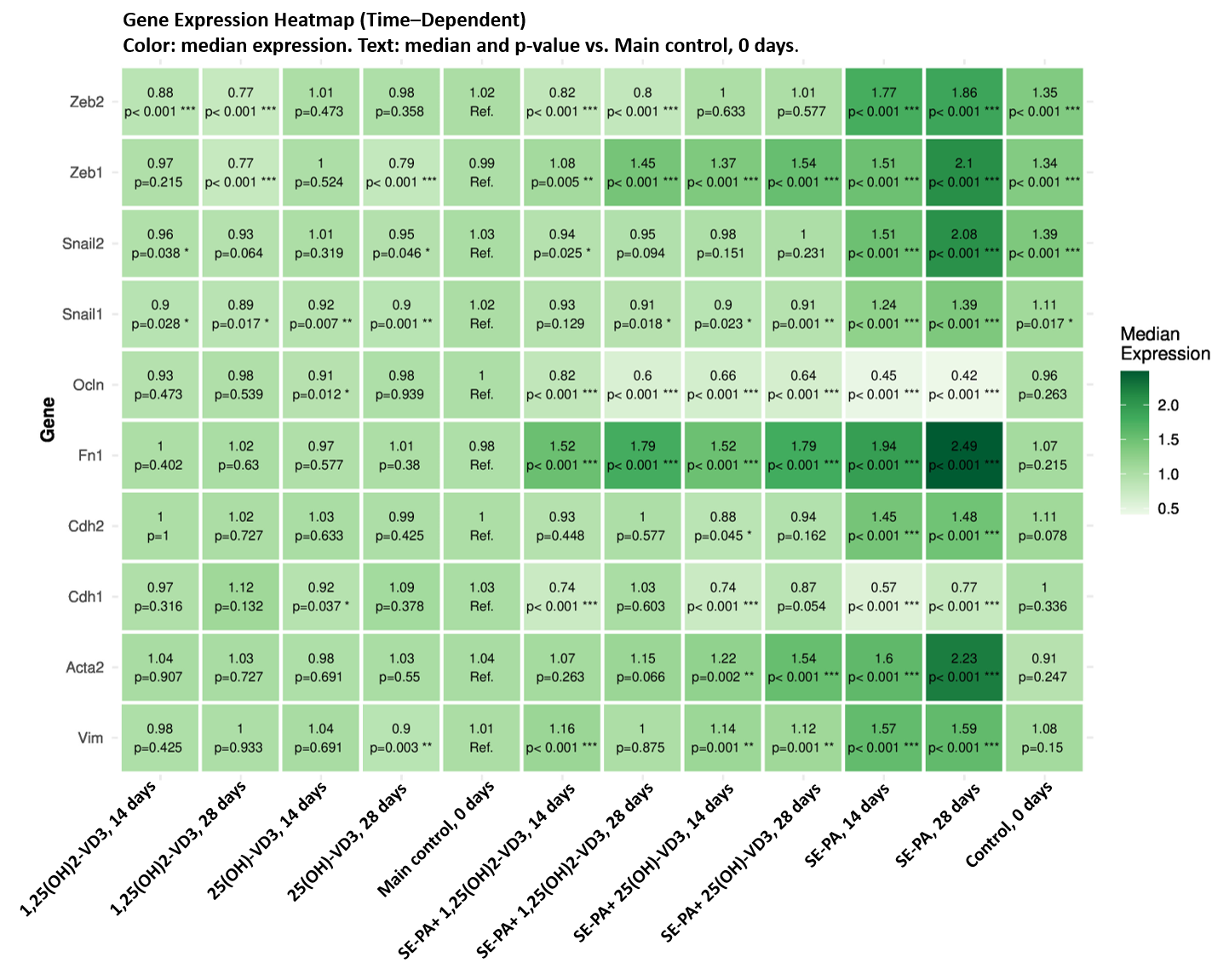 |
| --- |
| 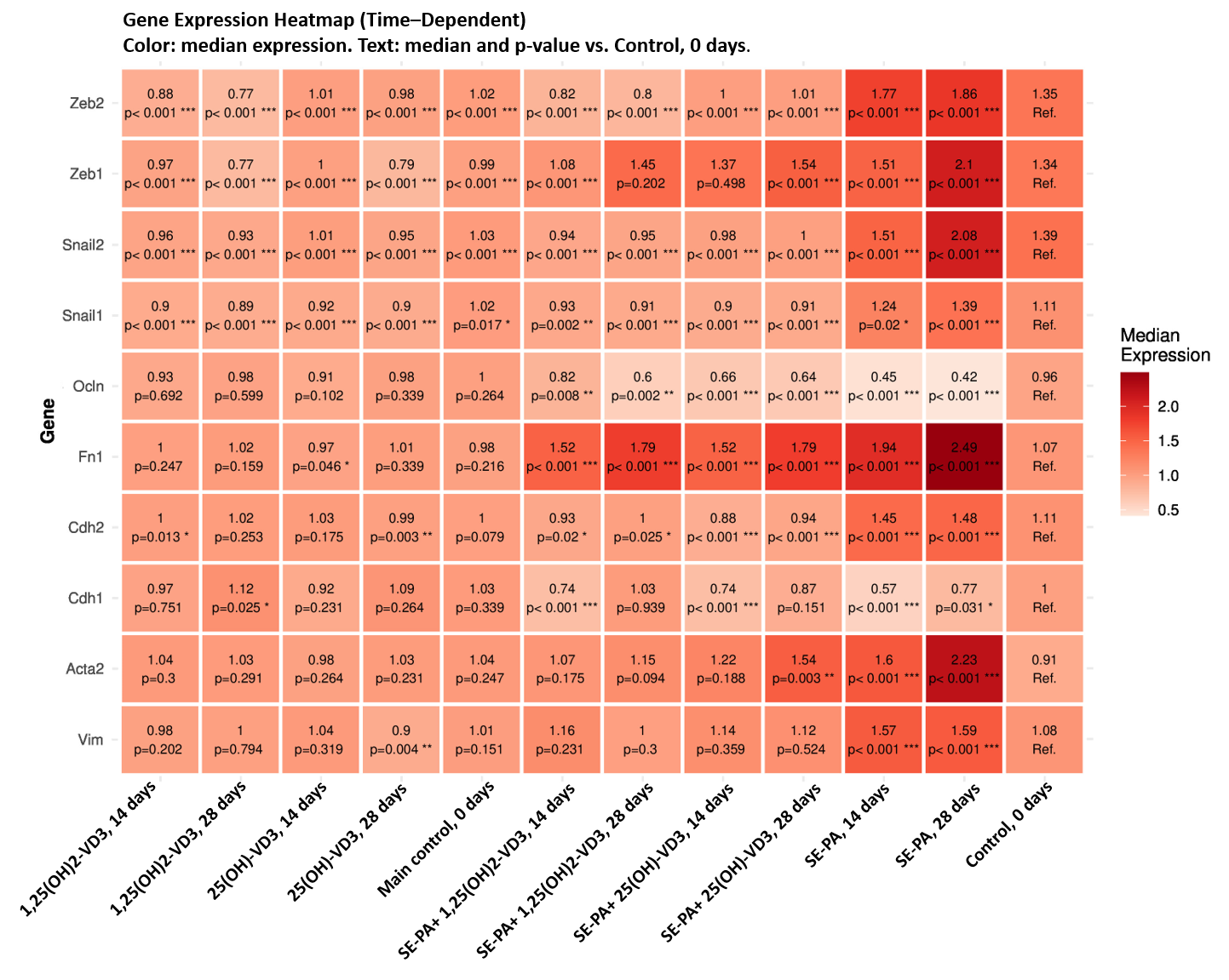 |
| 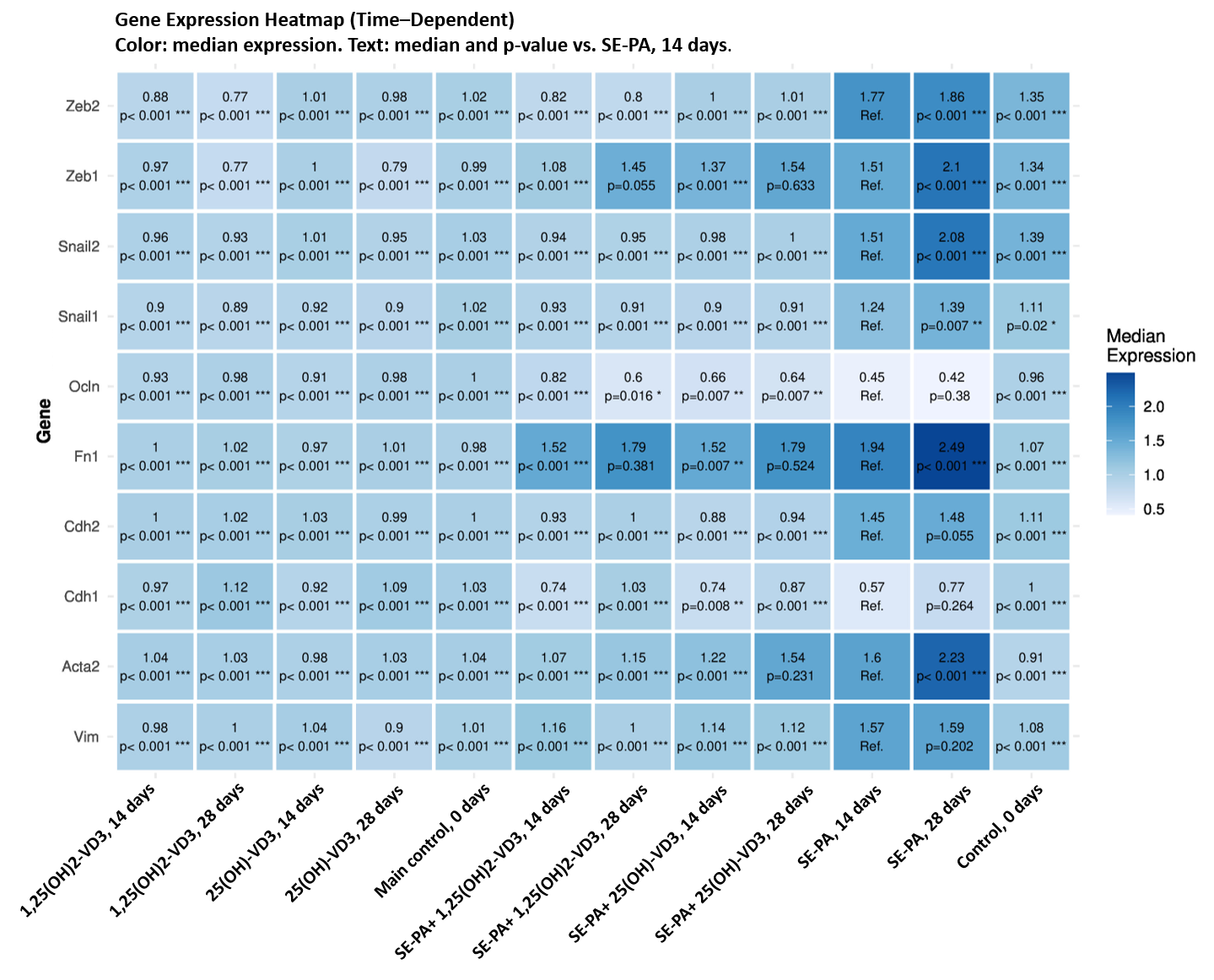 |
| 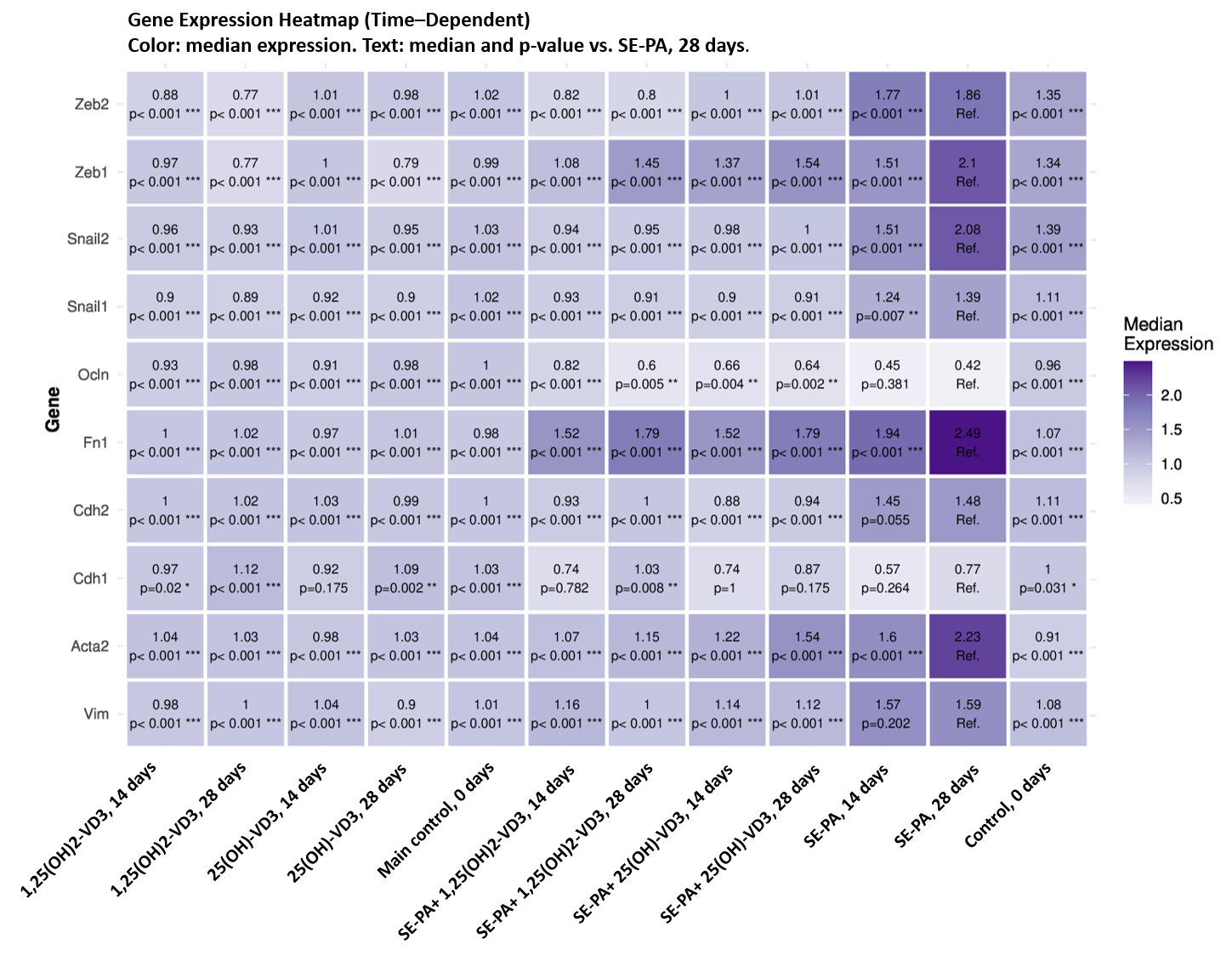 |
| **Figure S2**. Heatmap of the relative gene expression of EMT markers in the lungs of VD3-deficient mice inhaled with vitamin D3 metabolites and antigen of *Pantoea agglomerans*. Gene expression was investigated in lung homogenates using RealTime PCR methods. The heatmap displays the relative expression of selected genes (y-axis) across various experimental conditions and time points (x-axis). The color of each tile represents the median gene expression level for that condition, with the scale defined in the legend. Each tile is annotated with the numerical median value (top) and the statistical significance of the comparison (bottom). Statistical analysis was performed using the Wilcoxon rank-sum test. As the reference group were used as follows: Main Control 0 days, Control 0 days, SE-PA 14 days, SE-PA 28 days. Every experimental condition was compared against this single reference group. Significance levels are denoted as follows: *** for p < 0.001, ** for p < 0.01, * for p < 0.05, and ns for non-significant (p ≥ 0.05). |
